# Supplementary material for: Mechanistic Insights into Autoinhibition of the Oncogenic Chromatin Remodeler ALC1
Source: Mol Cell. 2017 Dec 7;68(5):847–859.e7. doi: 10.1016/j.molcel.2017.10.017 (PMC5745148; doi:10.1016/j.molcel.2017.10.017)
Supplement: Document S1. Figures S1–S7 and Tables S1 and S2 [file mmc1.pdf]

Molecular Cell, Volume *68*

Supplemental Information

Mechanistic Insights into Autoinhibition of the Oncogenic Chromatin Remodeler ALC1

Laura C. Lehmann, Graeme Hewitt, Shintaro Aibara, Alexander Leitner, Emil Marklund, Sarah L. Maslen, Varun Maturi, Yang Chen, David van der Spoel, J. Mark Skehel, Aristidis Moustakas, Simon J. Boulton, and Sebastian Deindl

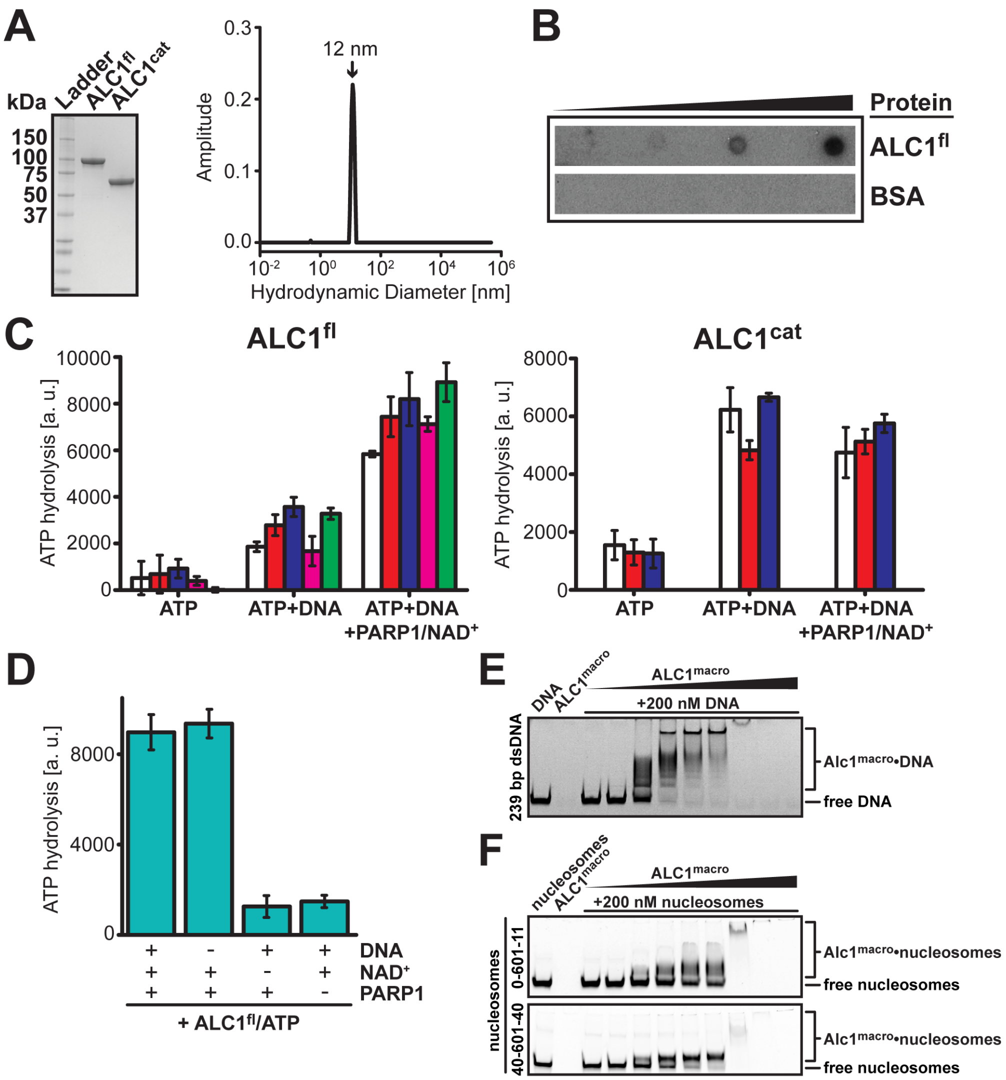

Figure S1. Related to Figure 1. Biochemical and biophysical characterization of ALC1.

(**A**) Left: SDS–PAGE analysis of ALC1^{fl} (~98.8 kDa; lacks only 14 residues at the N terminus and 18 residues at the C terminus) and ALC1^{cat} (~68.6 kDa; lacks the C-terminal macro domain and a portion of the linker, leaving only the ATPase motor and a 67-residue portion of the N terminus of the linker). Right: Dynamic light scattering (DLS) measurements with ALC1^{fl}. The size distribution by amplitude features a single peak at a hydrodynamic diameter of ~12 nm.

(**B**) Poly(ADP-ribose) binding assay for ALC1^{fl}. 2, 4, 8, and 16 pmol of ALC1^{fl} or BSA were dot-blotted onto a nitrocellulose membrane and incubated with ^{32}P-labeled PAR.

(**C**) Independent replicate experiments for ALC1^{fl} (left) and ALC1^{cat} (right) used to generate Figure 1A. Different colors represent the different repeats. Where indicated, PARP1/NAD^{+} and/or 239 bp double-stranded (ds) DNA were added. Error bars represent SEM (*N* = 3 technical replicates).

(**D**) Negative controls for the PARP1/NAD^{+} reaction in Figure 1A. All controls were conducted with ALC1^{fl}/ATP either in the presence or absence of PARP1, NAD^{+}, or 239 bp dsDNA. Error bars represent SEM (*N* = 3 independent experiments with 3 technical replicates each).

(**E**) Electrophoretic mobility of dsDNA (239 bp, 200 nM) in the absence or presence of 0.01, 0.1, 1, 1.3, 1.7, 2, 4, 8, and 16 µM ALC1 macro domain (ALC1^{macro}). As a comparison, lane 2 shows 16 µM ALC1^{macro} without dsDNA.

(**F**) Electrophoretic mobility of 200 nM FAM-labeled end-positioned (0-601-11; top gel) or center-positioned (40-601-40; bottom gel) nucleosomes in the absence or presence of 0.01, 0.1, 1, 1.3, 1.7, 2, 4, 8, and 16 µM ALC1^{macro}. As a comparison, lane 2 shows 16 µM ALC1^{macro} without nucleosomes. Both gels were imaged using FAM fluorescence.

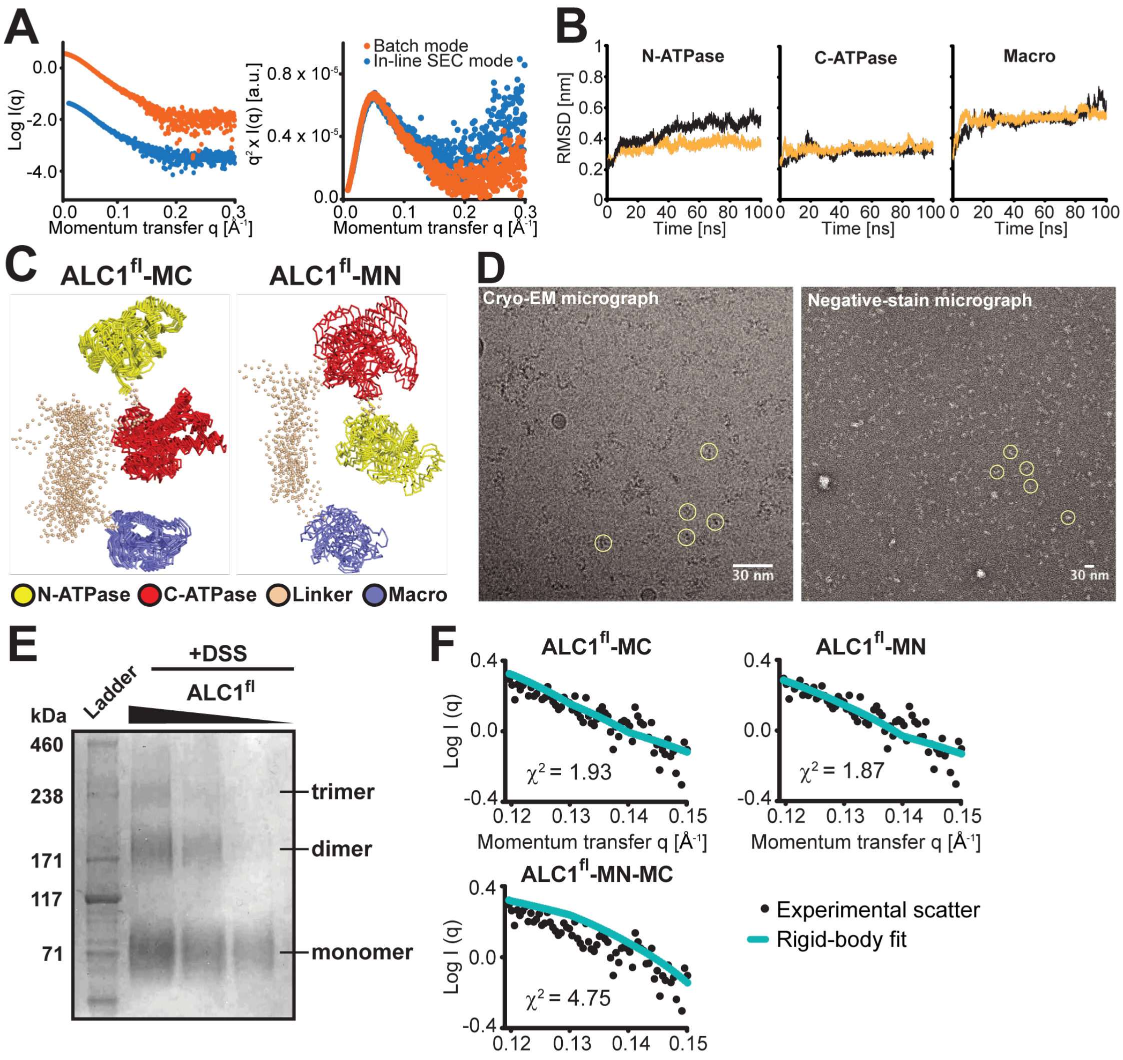

Figure S2. Related to Figure 2. Additional analyses for structural modeling of ALC1^{fl}.

(**A**) Left: Comparison of ALC1^{fl} SAXS data measured in batch mode (orange) or with in-line size exclusion chromatography (blue). Curves are shifted along the y-axis by an arbitrary offset. Right: Kratky plots.

(**B**) Molecular dynamics simulations of the homology models show that they are stable and without unnatural energetic strain. The structural fluctuations during duplicate (black and orange) 100 ns simulations for the N-terminal ATPase lobe (left), the C-terminal ATPase lobe (middle), and the macro domain (right) shown as root mean squared deviation (RMSD) time traces. RMSD values represent deviations from the input homology model of the protein backbone, calculated after superimposing the individual structures with the backbone of the entire homology model.

(**C**) Overlay of individual rigid-body models from the dominant clusters of similar ALC1^{fl}-MC (left) or ALC1^{fl}-MN (right) structures.

(**D**) Micrographs from cryo- (left) and negative-stain (right) EM.

(**E**) SDS–PAGE analysis of ALC1^{fl} cross-linking reactions with 1 mM DSS and 0.6, 0.3, and 0.15 mg/ml ALC1^{fl} (from left to right). At dilute concentrations, only monomeric ALC1^{fl} can be detected.

(**F**) Comparison of the experimental scattering recorded for ALC1^{fl} with the theoretical scattering calculated for representative rigid-body models obtained with Macro:C-ATPase interdomain cross-links only (top left), Macro:N-ATPase interdomain cross-links only (top right), or both Macro:N-ATPase and Macro:C-ATPase interdomain cross-links (bottom) as restraints. When Macro:N-ATPase and Macro:C-ATPase cross-links are used as restraints at the same time, rigid-body modeling yielded a substantially worse fit to the experimental scattering data.

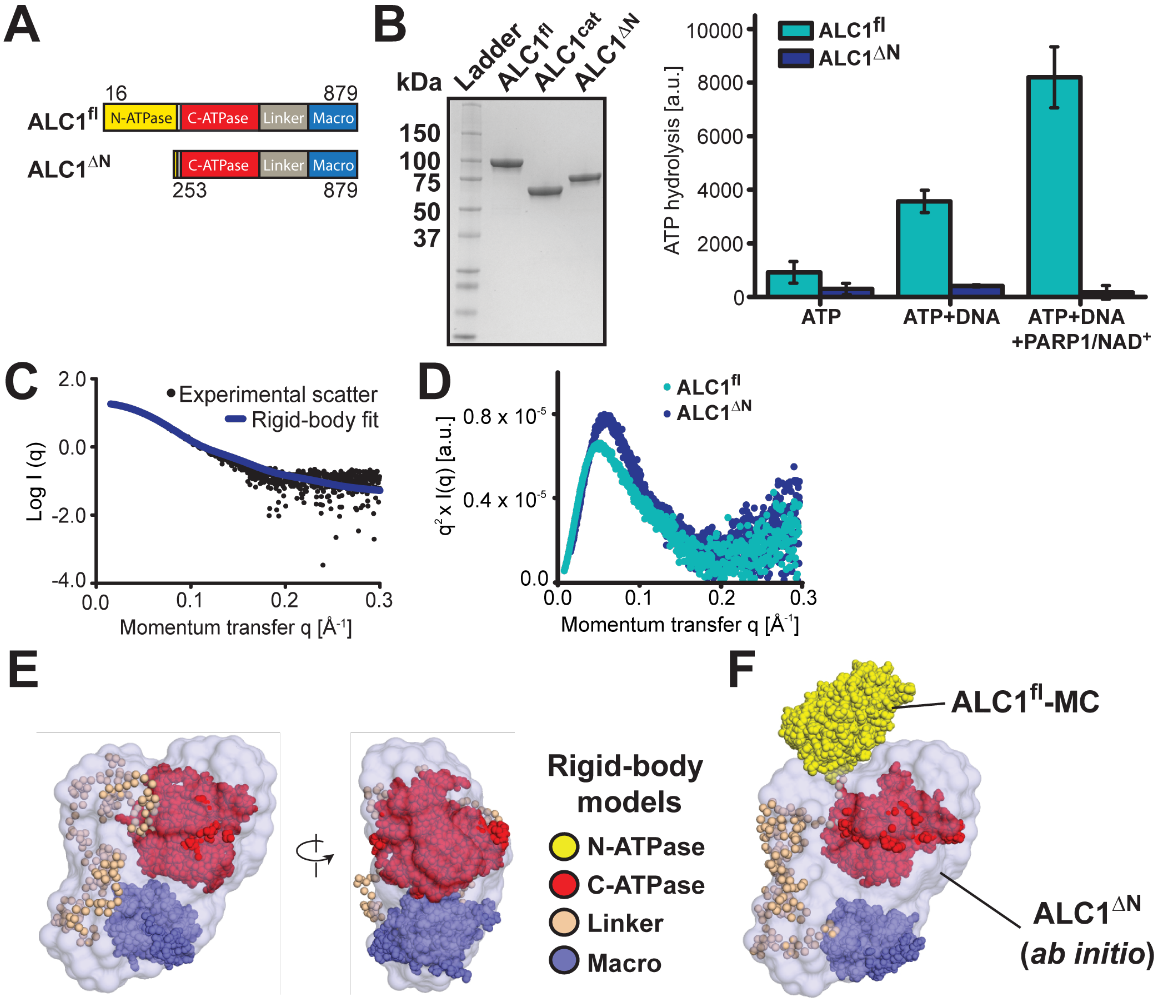

Figure S3. Related to Figure 2. ATPase activity and SAXS-based modeling of ALC1$^{\Delta N}$.
(**A**) Domain organization of ALC1^{fl} (top) and ALC1$^{\Delta N}$ (bottom).
(**B**) Left: SDS–PAGE analysis of ALC1$^{\Delta N}$ (71.7 kDa), ALC1^{fl} (98.8 kDa), and ALC1^{cat} (68.6 kDa). The same gel as in Figure S1A (where the last lane was cropped from the image) is shown. Right: Comparison of ATPase activities of ALC1$^{\Delta N}$ (dark blue) and ALC1^{fl} (teal). Where indicated, PARP1/NAD$^+$ and/or 239 bp dsDNA were added. Error bars represent SEM ($N = 3$ technical replicates).
(**C**) SAXS data for ALC1$^{\Delta N}$. The experimental data (black dots) are compared to the theoretical scattering curves (dark blue) calculated from the representative ALC1$^{\Delta N}$ rigid-body model shown (mean χ^2 =2.0 for all 20 independent rigid body modeling trials).
(**D**) Comparison of the Kratky plots derived from batch-mode SAXS data collected with ALC1^{fl} (teal) and ALC1$^{\Delta N}$ (dark blue).
(**E**) SAXS-derived structural model of ALC1$^{\Delta N}$. The structure obtained from rigid-body modeling with the C-terminal ATPase lobe, the linker, and the macro domain shown in red, light brown, and blue, respectively. The model is superimposed with a representative molecular envelope (gray) obtained from independent *ab initio* shape reconstruction.
(**F**) The ALC1^{fl}-MC structure is superimposed with the molecular envelope for ALC1$^{\Delta N}$ from (E).

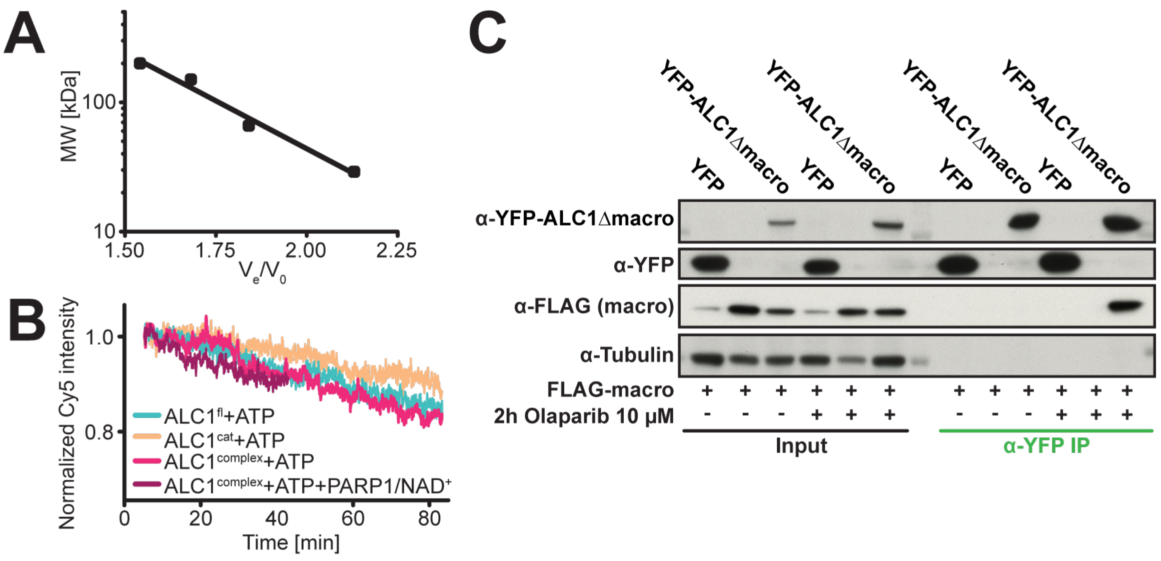

Figure S4. Related to Figure 4. *In vitro* and *in vivo* complex formation.

(**A**) Calibration curve for the analytical gel filtration shown in Figure 4A. β-amylase (200 kDa), alcohol dehydrogenase (150 kDa), bovine serum albumin (66 kDa), and carbonic anhydrase (29 kDa) were used as molecular weight standards to calibrate the Superdex 200 10/300 GL column.

(**B**) Nucleosome remodeling time course for $ALC1^{complex}$ in the presence of ATP (pink) compared to $ALC1^{fl}$ (teal) and $ALC1^{cat}$ (orange). Additionally, a remodeling time course for $ALC1^{complex}$ in the presence of ATP and PARP1/NAD^+ is shown. PARP1/NAD^+ releases the macro domain from the ATPase motor (Figure 4B), resulting in virtually the same remodeling kinetics as those observed with $ALC1^{cat}$. The curve for $ALC1^{fl}$ is the same as in Figure 1C.

(**C**) Co-immunoprecipitation (coIP) assay of U2OS cells transfected with plasmids expressing FLAG-tagged macro domain (FLAG-macro), YFP, or an YFP-tagged version of the ATPase motor (YFP-ALC1Δmacro). Cells were grown in the presence or absence of olaparib, as indicated. Cell lysates were immunoprecipitated with α-YFP (reciprocal to Figure 4C). The cell lysates (input) and immunoprecipitates were analyzed by immunoblotting with antibodies as indicated.

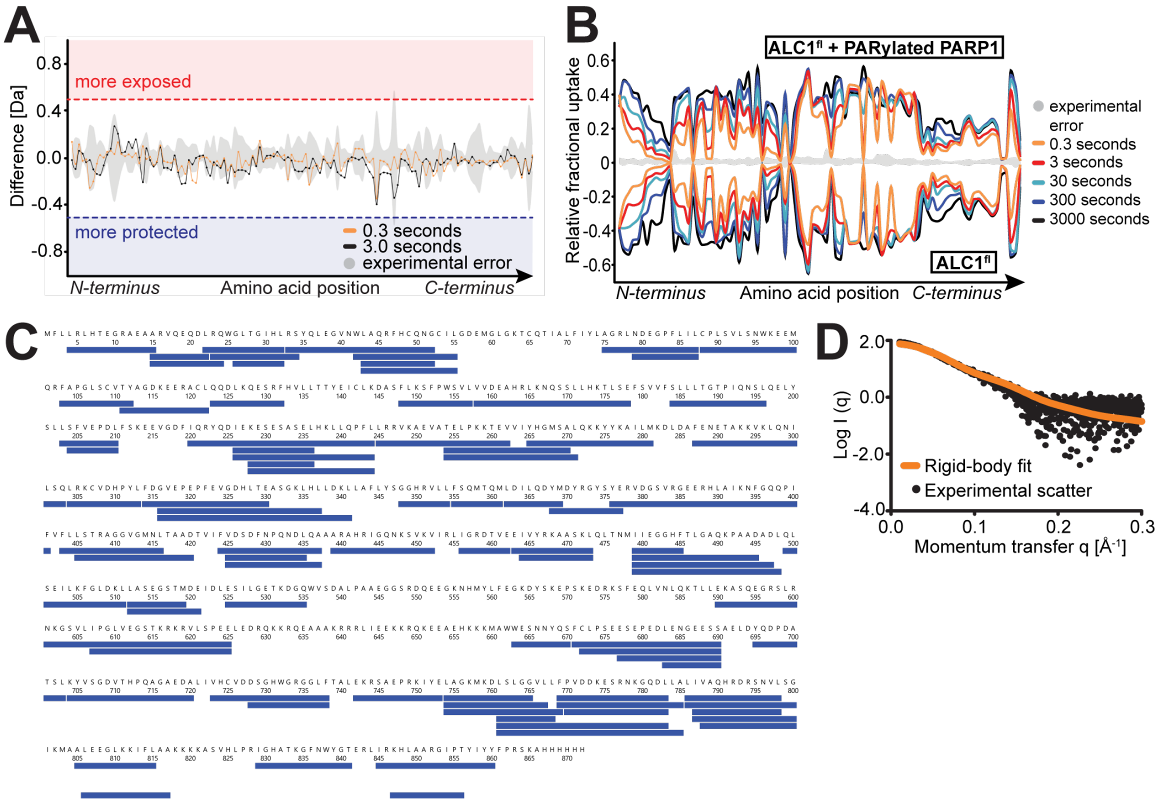

Figure S5. Related to Figure 5. Additional HDX-MS analyses of ALC1^{fl} and SAXS measurements with ALC1^{cat}.

(**A**) Difference plot of hydrogen-deuterium exchange (HDX) data from ALC1^{fl} in the presence and absence of non-PARylated PARP1. Changes beyond a threshold of ± 0.5 fall into areas of increased exposure (positive difference, shaded red) or protection (negative difference, shaded blue). The experimental error is shown in grey. Different line colors indicate the different time points at which deuterium labeling was carried out.

(**B**) Butterfly representation comparing the relative deuterium uptake of different regions of ALC1^{fl} in the presence of PARylated PARP1 (top) with that of ALC1^{fl} alone (bottom). The plot is shown for 5 different D_2O incubation times (indicated by different colors) together with the experimental error from three replicates in grey. The difference plot shown in Figure 5A is derived from the data shown in the butterfly plot.

(**C**) Sequence coverage of ALC1^{fl} for HDX experiments. The coverage is 75.2% with a total of 88 detected peptides.

(**D**) SAXS data measured with ALC1^{cat} (black dots) and comparison with the theoretical scattering curves (orange) calculated from the rigid-body model shown in Figure 5C.

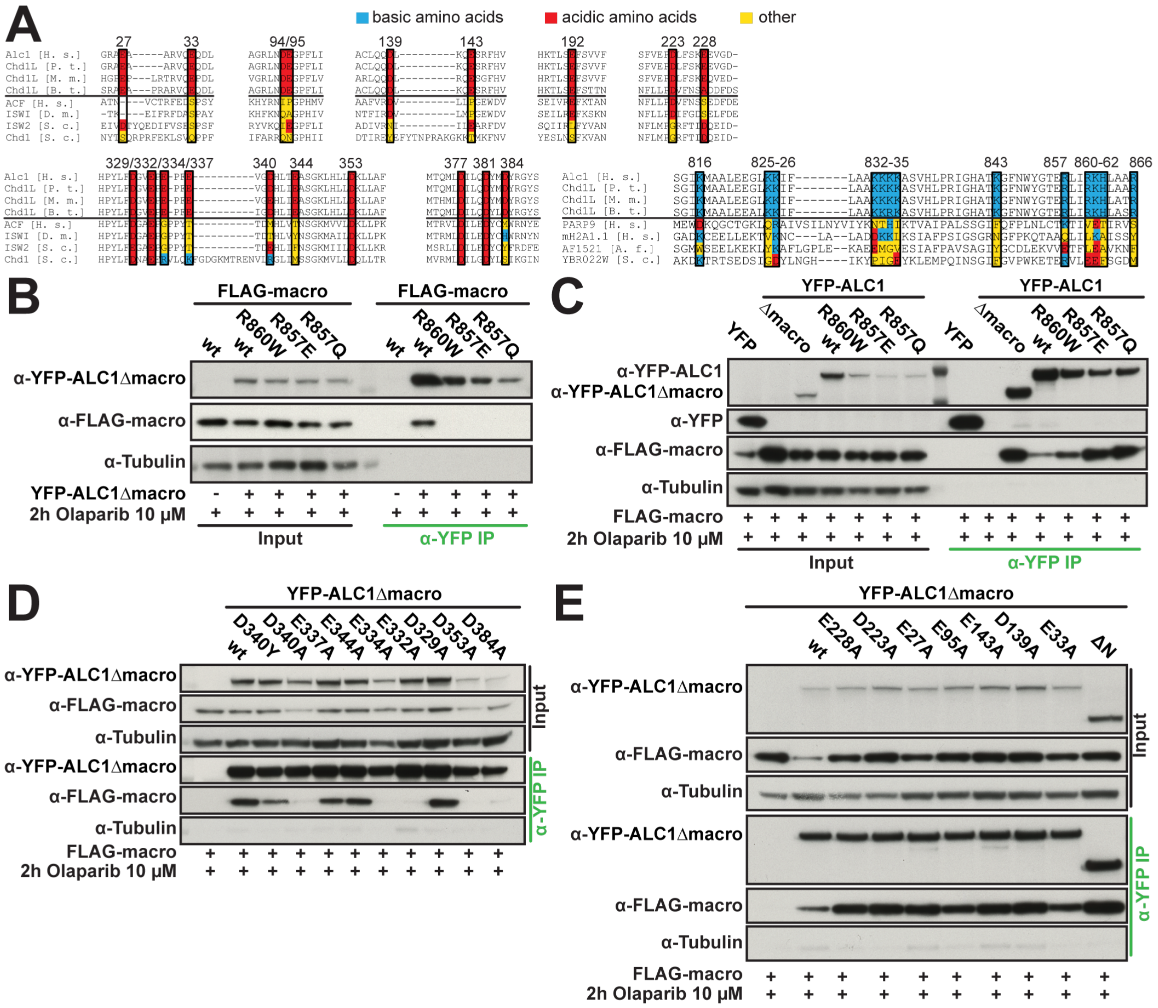

Figure S6. Related to Figure 6. Sequence alignments and reciprocal co-IP assays.

(**A**) Sequence alignments for the N- (top) or C-terminal (bottom left) ATPase lobes from ALC1/CHD1L and other remodelers and for the macro domains from ALC1/CHD1L and other proteins (bottom right). H.s. - *Homo sapiens*; P. t. - *Pan troglodytes*; M. m. - *Mus musculus*; B. t. - *Bos taurus*; D. s.: *Drosophila melanogaster*; S. c.: *Saccharomyces cerevisiae*; A. f. - *Archaeoglobus fulgidus*. Basic/acidic amino acids are highlighted in blue/red, respectively; others in yellow. The residue numbering corresponds to ALC1 [H. s.].

(**B-E**) CoIP assays with U2OS cells to probe macro domain-ATPase motor interactions. Cell lysates were immunoprecipitated with α-YFP (reciprocal to Figures 6B-E). As indicated, cells were grown and transfected with plasmids described for Figure 6. α-YFP immunoprecipitates were analyzed by immunoblotting with antibodies as indicated.

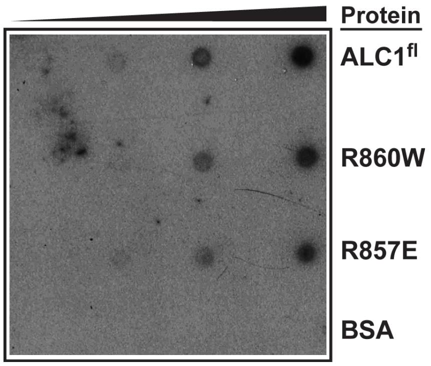

Figure S7. Related to Figure 7. Binding of poly(ADP-ribose) (PAR) to wild-type ALC1^{fl} and to ALC1^{fl} mutants.

2, 4, 8, and 16 pmol of ALC1^{fl} wild-type, R860W, R857E, and BSA were dot-blotted onto a nitrocellulose membrane and incubated with ^{32}P-labeled PAR. The same dot-blot as in Figure S1B (where the ALC1^{fl} mutants R860W and R857E were cropped from the image) is shown.

Peptides	Residue 1	Residue 2	Id-Score
KTEVVIYHGMSALQK-RVKAEVATELPK-a1-b3	273	263	32.67
KTEVVIYHGMSALQKK-VKAEVATELPK-a1-b2	273	263	29.01
KTEVVIYHGMSALQK-VKAEVATELPK-a1-b2	273	263	28.71
ACLQQDLKQESR-IGQNKSVK-a8-b5	141	462	29.58
LKNQSSLLHK-IGQNKSVK-a2-b5	180	462	27.81
YQDIEKESESASELHK-KAASK-a6-b1	242	482	30.07
YQDIEKESESASELHK-SVKVIR-a6-b3	242	465	33.00
YQDIEKESESASELHK-IGQNKSVK-a6-b5	242	462	30.08
VKAEVATELPK-SVKVIR-a2-b3	263	465	30.81
RVKAEVATELPK-SVKVIR-a3-b3	263	465	29.08
GGLFTALEKR-LKNQSSLLHK-a9-b2	757	180	28.60
NKGQDLLALIVAQHR-ACLQQDLKQESR-a2-b8	793	141	24.49
NKGQDLLALIVAQHR-LKNQSSLLHK-a2-b2	793	180	28.82
IGHATKGFNWYGTER-ACLQQDLKQESR-a6-b8	848	141	29.58
FAPGLSCVTYAGDKEER-KHLAAR-a14-b1	130	861	26.65
FAPGLSCVTYAGDKEER-NKGQDLLALIVAQHR-a14-b2	130	793	16.52
FAPGLSCVTYAGDKEER-GGLFTALEKR-a14-b9	130	757	26.14
ACLQQDLKQESR-GGLFTALEKR-a8-b9	141	757	29.92
LKNQSSLLHK-KHLAAR-a2-b1	180	861	27.95
YQDIEKESESASELHK-KHLAAR-a6-b1	242	861	28.33
YQDIEKESESASELHK-IGHATKGFNWYGTER-a6-b6	242	848	29.70
YQDIEKESESASELHK-NKGQDLLALIVAQHR-a6-b2	242	793	18.20
YQDIEKESESASELHK-GGLFTALEKR-a6-b9	242	757	24.01
VKAEVATELPK-KHLAAR-a2-b1	263	861	26.30
VKAEVATELPK-GGLFTALEKR-a2-b9	263	757	25.92
GGLFTALEKR-IGQNKSVK-a9-b5	757	462	28.39
GGLFTALEKR-SVKVIR-a9-b3	757	465	31.50
SNVLSGIKMAALEEGLKK-IGQNKSVKVIR-a8-b8	816	465	23.75
IGHATKGFNWYGTER-SVKVIR-a6-b3	848	465	30.79
IGQNKSVK-KIYELAGK-a5-b1	462	764	24.41

Table S1. Related to Figure 2. List of cross-linked peptides.

Inter-domain cross-links between the N-terminal and the C-terminal ATPase lobes, between the macro domain and the N-terminal ATPase lobe, and between the macro domain and the C-terminal ATPase lobe are shown in orange, green, and purple, respectively. Only cross-links with an Id score (Walzthoeni et al., 2012) of > 15 were included.

Construct/Mutation	Forward Primer	Reverse Primer
ALC1Δmacro	TAAACAAAGTGGTGATATCAAAC	TTCCTTTTGCCTCTTCTTC
ALC1ΔN	GAGGTAGCTACAGAGCTTC	GAAGCCTGCTTTTTTGTAC
D94A	CCAGCTGGAGgccGAGTAAACTG	TAAGAGCGTAGGTGAATC
E228A	CTTTTCCAAGgccGAGGTGGGAG	AGATCAGGCTCCACAAAAC
D223A	TGTGGAGCCTgccCTCTTTTCCAAG	AAACTGAGGAGGGAGTAG
E27A	GGGCCGAGCCgccGCGGCGCGGG	GCCGCCTCGGCTCGGCCC
E192A	GACCTTGTCAgccTTCTCAGTAGTCTTCAGTC	TTATGCAGCAGGGAGCTT
E95A	ATTAAATGATgccGGGCCATTTCTG	CTTCCTGCCAAATAAATG
E143A	CCTGAAACAGgccTCACGTTTTCATGTGCTAC	TCTTGCTGAAGGCAGGCT
D139A	CCTTCAGCAAgccCTGAAACAGG	CAGGCTCTTTCCTCCTTG
E33A	GCGGGTGCAGgccCAGGACTTACGGCAGTGGG	GCCGCCTCGGCTCGGCCC
D340Y	TGAAGTTGGAtacCACCTGACTGAGG	AAAGGCTCCGGCTCCACA
D340A	TGAAGTTGGAgccCACCTGACTG	AAAGGCTCCGGCTCCACA
E337A	GGAGCCTTTTgccGTTGGAGACC	GGCTCCACACCATCAAAC
E344A	CCACCTGACTgccGCTAGTGGGAAG	TCTCCAACTTCAAAAGGC
E334A	TGTGGAGCCGgccCCTTTTGAAG	CCATCAAACAAATATGGGTG
E332A	TGATGGTGTGgccCCGGAGCCTT	AACAAATATGGGTGATCCAC
D329A	ATATTTGTTTgccGGTGTGGAGCCG	GGGTGATCCACACACTTTC
D353A	TCACCTGCTGgccAAGCTACTAGC	AGCTTCCCACTAGCCTCA
D384A	GACTATATGGgccACAGAGGCTACAG	TTGGAGAATATCCAACATC
R857Q	TGGTACTGAGcaaCTTATTCGGAAAC	TACCAGTTAAAACCTTTCGTG
R857E	TGGTACTGAGgaaCTTATTCGGAAAC	TACCAGTTAAAACCTTTCG
R860W	GCGACTTATTtggAAACATCTGG	TCAGTACCATACCAGTTAAAAC

Table S2. Related to STAR Methods. DNA oligonucleotides used to create ALC1 constructs for *in vivo* analysis.
