# Supplementary material for: Mechanistic Insights into Autoinhibition of the Oncogenic Chromatin Remodeler ALC1
Source: Mol Cell. 2017 Dec 7;68(5):847–859.e7. doi: 10.1016/j.molcel.2017.10.017 (PMC5745148; doi:10.1016/j.molcel.2017.10.017)
Supplement: Document S2. Article plus Supplemental Information [file mmc2.pdf]

Article

Molecular Cell

# Mechanistic Insights into Autoinhibition of the Oncogenic Chromatin Remodeler ALC1

## Graphical Abstract

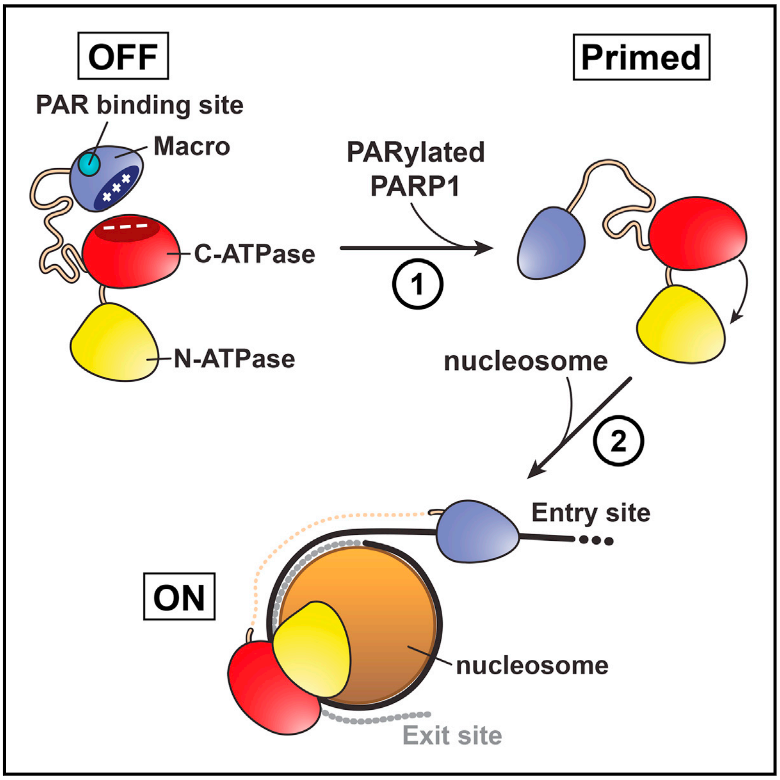


## Highlights

- ALC1 macro domain facilitates remodeling and autoinhibits ATPase activity
- A macro domain/ATPase interface maintains catalytic autoinhibition
- Interface mutations activate the ATPase and alter recruitment at DNA damage sites
- PARylated PARP1 binding triggers a conformational change that activates ALC1

## Authors

Laura C. Lehmann, Graeme Hewitt, Shintaro Aibara, ...,
Aristidis Moustakas, Simon J. Boulton, Sebastian Deindl

## Correspondence

simon.boulton@crick.ac.uk (S.J.B.), sebastian.deindl@icm.uu.se (S.D.)

## In Brief

Lehmann et al. employ an integrative structural approach with biochemistry, cell-based assays, and *in vivo* imaging to investigate the regulation of ALC1. An unappreciated functional crosstalk between the PAR-binding macro domain and the ATPase underlies autoinhibition and activation of ALC1.

Lehmann et al., 2017, Molecular Cell *68*, 847–859
December 7, 2017 © 2017 The Authors. Published by Elsevier Inc.
https://doi.org/10.1016/j.molcel.2017.10.017

CellPress

# Mechanistic Insights into Autoinhibition of the Oncogenic Chromatin Remodeler ALC1

Laura C. Lehmann,[1,8] Graeme Hewitt,[2,8] Shintaro Aibara,[3] Alexander Leitner,[4] Emil Marklund,[1] Sarah L. Maslen,[5] Varun Maturi,[6] Yang Chen,[1] David van der Spoel,[7] J. Mark Skehel,[5] Aristidis Moustakas,[6] Simon J. Boulton,[2,*] and Sebastian Deindl[1,9,*]
[1]Department of Cell and Molecular Biology, Science for Life Laboratory, Uppsala University, 75124 Uppsala, Sweden
[2]The Francis Crick Institute, 1 Midland Road, London NW1 1AT, UK
[3]Science for Life Laboratory, Department of Biochemistry and Biophysics, Stockholm University, 17165 Solna, Sweden
[4]Department of Biology, Institute of Molecular Systems Biology, Swiss Federal Institute of Technology, 8093 Zürich, Switzerland
[5]MRC Laboratory of Molecular Biology, Francis Crick Avenue, Cambridge Biomedical Campus, Cambridge CB2 0QH, UK
[6]Department of Medical Biochemistry and Microbiology, and Ludwig Institute for Cancer Research, Science for Life Laboratory, Uppsala University, 75123 Uppsala, Sweden
[7]Department of Cell and Molecular Biology, Computational Biology and Bioinformatics, Uppsala University, 75124 Uppsala, Sweden
[8]These authors contributed equally
[9]Lead Contact
*Correspondence: simon.boulton@crick.ac.uk (S.J.B.), sebastian.deindl@icm.uu.se (S.D.)
https://doi.org/10.1016/j.molcel.2017.10.017

## SUMMARY

**Human ALC1 is an oncogene-encoded chromatin-remodeling enzyme required for DNA repair that possesses a poly(ADP-ribose) (PAR)-binding macro domain. Its engagement with PARylated PARP1 activates ALC1 at sites of DNA damage, but the underlying mechanism remains unclear. Here, we establish a dual role for the macro domain in autoinhibition of ALC1 ATPase activity and coupling to nucleosome mobilization. In the absence of DNA damage, an inactive conformation of the ATPase is maintained by juxtaposition of the macro domain against predominantly the C-terminal ATPase lobe through conserved electrostatic interactions. Mutations within this interface displace the macro domain, constitutively activate the ALC1 ATPase independent of PARylated PARP1, and alter the dynamics of ALC1 recruitment at DNA damage sites. Upon DNA damage, binding of PARylated PARP1 by the macro domain induces a conformational change that relieves autoinhibitory interactions with the ATPase motor, which selectively activates ALC1 remodeling upon recruitment to sites of DNA damage.**

## INTRODUCTION

Accessibility to the DNA during many vital cellular transactions is regulated in part by ATP-dependent chromatin-remodeling enzymes (remodelers) (Bartholomew, 2014; Becker and Workman, 2013; Bowman, 2010; Clapier and Cairns, 2009; Narlikar et al., 2013). These remodelers typically possess a catalytic subunit that encompasses a conserved Snf2 family (sucrose non-fermenter 2) ATPase and flanking domains that can give rise to distinct remodeling outcomes (Flaus et al., 2006; Narlikar et al., 2013).

Human ALC1 (Amplified in liver cancer 1), also known as CHD1L (Chromodomain-helicase-DNA-binding protein 1-like), is a remodeler encoded by a gene located within human chromosome 1q21, a region that is frequently amplified in hepatocellular carcinomas (Ma et al., 2008; Marchio et al., 1997; Wong et al., 2003). Evidence suggests that ALC1 is a driver of oncogenesis (Chen et al., 2009, 2010; Ma et al., 2008; Mu et al., 2015). Additionally, ALC1 is involved in DNA damage repair (Ahel et al., 2009; Gottschalk et al., 2009, 2012; Pines et al., 2012; Sellou et al., 2016).

ALC1 is unique among remodelers in that it possesses a macro domain that can selectively bind to poly(ADP-ribose) (PAR) (Ahel et al., 2009; Gottschalk et al., 2009) synthesized by PAR polymerases (PARPs) at DNA damage sites (Hassa et al., 2006; Lindahl et al., 1995; Satoh and Lindahl, 1992). PARylation is associated with chromatin relaxation (de Murcia et al., 1986; Frechette et al., 1985; Leduc et al., 1986; Poirier et al., 1982), which is believed to facilitate DNA repair (D'Amours et al., 1999; El-Khamisy et al., 2003; Malanga and Althaus, 2005; Realini and Althaus, 1992). The catalytic activity of ALC1 is negligible until activated by PARP1 and its substrate $NAD^+$ (Ahel et al., 2009; Gottschalk et al., 2009). Engagement of the macro domain with the PAR chains on PARylated PARP1 has been suggested to allosterically stimulate ALC1 catalytic activity (Gottschalk et al., 2012). However, the molecular basis for such a mechanism remains elusive.

The ATPase of Snf2 proteins including ALC1 comprises two RecA-like domains (Singleton et al., 2007) (N-terminal and C-terminal ATPase lobes) that associate to adopt a closed, active conformation (Hauk and Bowman, 2011; Hopfner et al., 2012; Narlikar et al., 2013) as observed for RAD54 (Thomä et al., 2005). In the structure of the Snf2 homolog SSO1653, however, the two lobes are flipped 180° when compared to the closed conformation (Dürr et al., 2005). The structures of *S. cerevisiae* CHD1 (Hauk et al., 2010) and of *M. thermophila* SNF2 (Xia

 © 2017 The Authors. Published by Elsevier Inc. 
This is an open access article under the CC BY license (http://creativecommons.org/licenses/by/4.0/).

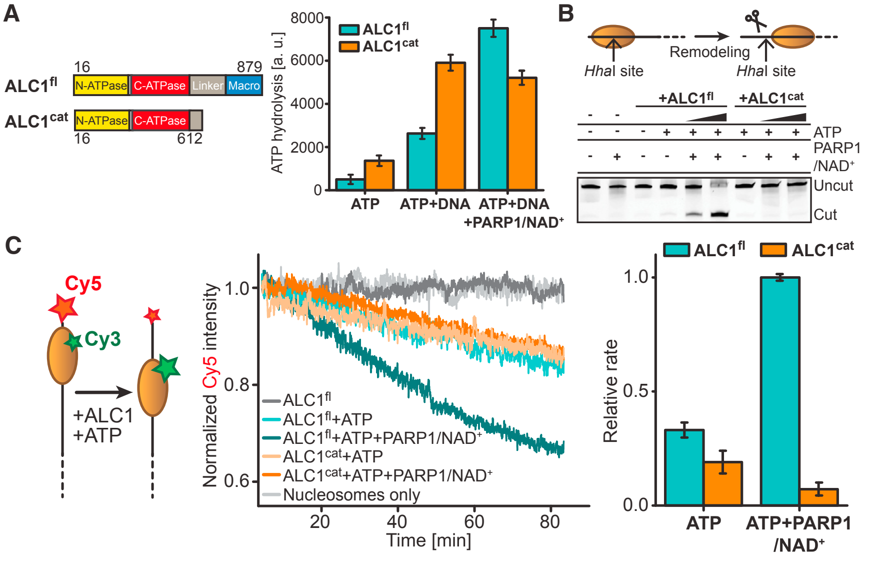


**Figure 1. Catalytic Activities of ALC1$^{fl}$ and ALC1$^{cat}$**

(A) Left: domain organization of ALC1$^{fl}$ and ALC1$^{cat}$. Right: ATPase activities of ALC1$^{fl}$ (teal) and ALC1$^{cat}$ (orange).

(B) Nucleosome remodeling by ALC1$^{fl}$ and ALC1$^{cat}$. The intrinsic HhaI cut site is initially protected by the histone octamer but becomes accessible upon ALC1-induced remodeling.

(C) Time courses for remodeling by ALC1$^{fl}$ and ALC1$^{cat}$ in the presence or absence of PARP1/NAD$^+$. Bar graph: relative remodeling rates for ALC1$^{fl}$ (teal) and ALC1$^{cat}$ (orange).

Error bars ± SEM. See also Figure S1.

et al., 2016) revealed an inactive conformation with splayed-apart ATPase lobes. In a recent structure of *M. thermophila* ISWI, autoinhibitory elements stabilize an inactive conformation (Yan et al., 2016). In these examples, the corresponding ATPase lobes exhibit extremely similar structures. However, in terms of their relative orientation, the two ATPase lobes can adopt various distinct conformations to regulate catalytic activity. For the oncogene-encoded human remodeler ALC1, there is no structural information available. Importantly, the molecular basis for the regulation of catalytic activity of ALC1 by its macro domain, which makes ALC1 unique among remodelers, remains unclear.

Here, we identify two distinct roles for the macro domain of ALC1, in the autoinhibition of ATPase activity and in its coupling to nucleosome mobilization. We establish that ALC1 can adopt two distinct conformations that differ only in the position of the macro domain against either the N- or C-terminal ATPase lobe. In either conformation, the ATPase lobes are held apart in an open configuration that is incompatible with catalytic activity. This inactive conformation is maintained by the placement of the macro domain against the ATPase motor via interactions between basic residues of the macro domain and acidic residues of the ATPase. Indeed, mutation of these residues displaces the macro domain from the ATPase, constitutively activates ALC1 independently of PARylated PARP1, and compromises the timely recruitment and disassembly of ALC1 from damage sites *in vivo*. Upon binding PARylated PARP1, the macro domain of ALC1 is displaced from its ATPase, which we propose is a key step in the selective activation of ALC1 upon recruitment to DNA damage sites.

## RESULTS

### Macro Domain Removal Abolishes the Dependence of ALC1 on Activation by PARylated PARP1

To investigate the regulatory role of the ALC1 macro domain, we purified an essentially full-length construct of human ALC1 (ALC1$^{fl}$, Figures 1A, S1A, and S1B) and a variant that lacks the macro domain (ALC1$^{cat}$, Figures 1A and S1A). ATPase measurements revealed that the activity of ALC1$^{fl}$ increased ~5.2-fold with double-stranded DNA (dsDNA) and was further enhanced ~2.9-fold upon incubation with PARP1/NAD$^+$ (Figures 1A, S1C, and S1D). Similarly, DNA augmented the ATPase activity of ALC1$^{cat}$ by ~4.3-fold. However, no further stimulation of ALC1$^{cat}$ ATPase activity was detected with PARP1/NAD$^+$. Since ALC1$^{cat}$ exhibited an ~2.7- and ~2.2-fold higher ATPase activity in the absence or presence of DNA, respectively, compared to ALC1$^{fl}$, these results raise the possibility that the macro domain acts to autoinhibit ALC1 ATPase under non-damaging conditions.

Next, we examined remodeling by ALC1 using nucleosomes with an initially protected HhaI site. Restriction enzyme accessibility was slightly increased upon incubation with ALC1$^{fl}$/ATP and substantially elevated by the addition of PARP1/NAD$^+$ (Figure 1B), consistent with previous findings (Gottschalk et al., 2009). Despite its elevated ATPase activity, ALC1$^{cat}$ did not appreciably remodel nucleosomes under any condition. We also employed a fluorescence resonance energy transfer (FRET) assay (Yang et al., 2006) to monitor ALC1-catalyzed remodeling, in which nucleosomes are labeled on one end of the linker DNA with the FRET acceptor Cy5 and on histone H2A with the FRET donor Cy3 (Figure 1C). Upon addition of ALC1$^{fl}$/ATP, FRET decreased as a result of nucleosome remodeling. The remodeling rate was increased by ~3-fold with PARP1/NAD$^+$. In contrast, ALC1$^{cat}$ exhibited dramatically reduced remodeling rates either with or without PARP1/NAD$^+$. We therefore considered a possible role of the macro domain as a substrate-binding element during remodeling. Indeed, electrophoretic mobility shift assays indicate that the macro domain binds both linker DNA and nucleosomes with ~1- to 2-μM affinity (Figures S1E and S1F). Hence, the macro domain is important for nucleosome remodeling and also acts to autoinhibit the ATPase under non-activating conditions.

### SAXS Reveals the Position of the Macro Domain against the ATPase under Non-activating Conditions

To gain insight into the structure of ALC1 under non-activating conditions, we conducted small-angle X-ray scattering (SAXS) measurements (Figures 2A and 2C), which revealed a well-folded,

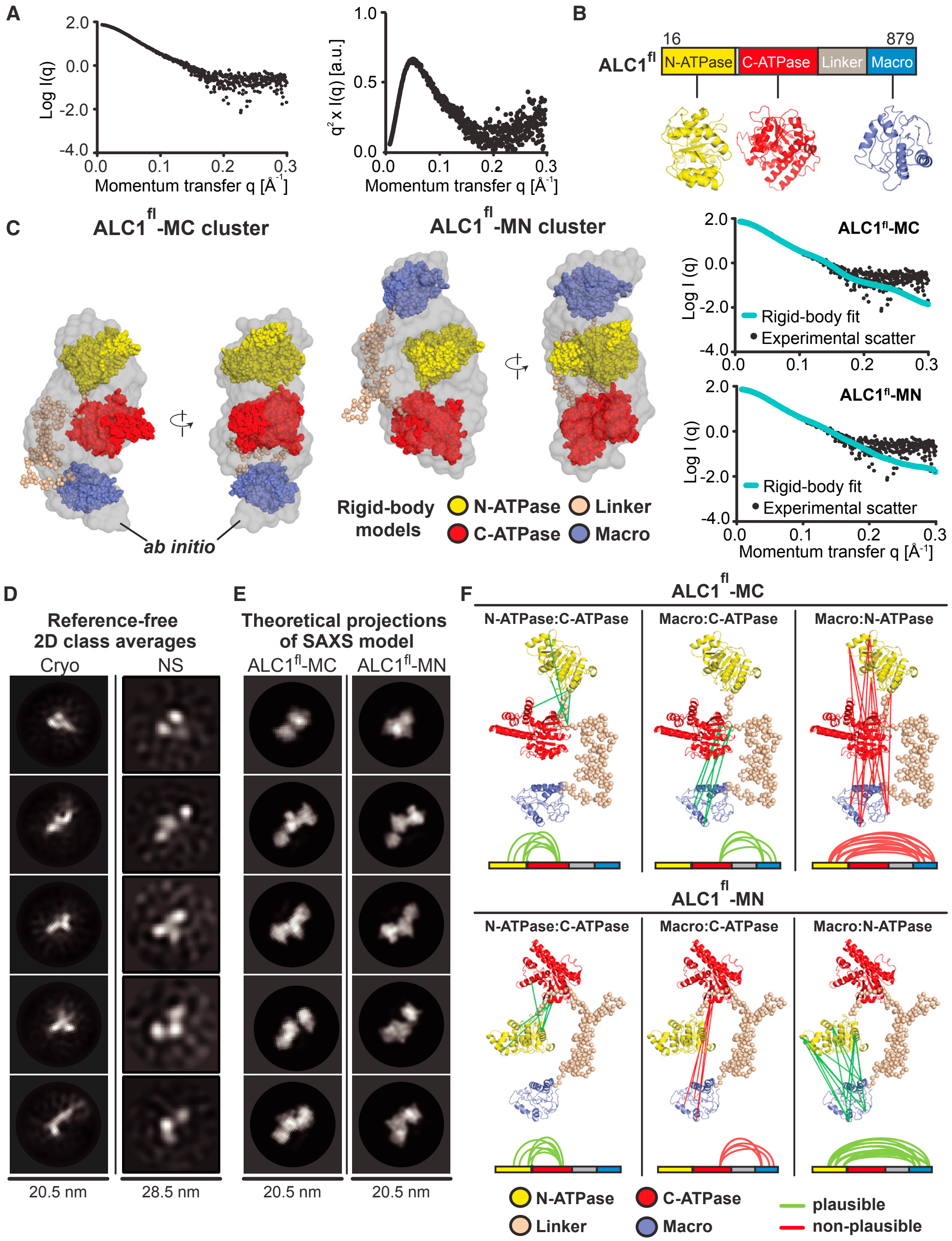


**Figure 2. Structural Analyses of ALC1fl**

(A) Left: SAXS data for ALC1fl. Right: same data plotted as $q^2 \times I$ versus q.

(B) Domain homology models.

(C) Representative SAXS-derived rigid-body models of ALC1fl (left: ALC1fl-MC, right: ALC1fl-MN), superimposed with the same representative *ab initio* molecular envelope (gray). Comparisons of the experimental (black dots) with the theoretical scattering (teal) are shown in the right panel.

(D) 2D class averages from cryo- and negative stain ("NS") EM.

*(legend continued on next page)*

monodisperse, and monomeric ALC1$^{fl}$ protein with a radius of gyration ($R_g$) of ~39.8 Å (Figures 2A and S2A). We carried out two types of SAXS-based modeling approaches (Hura et al., 2009; Petoukhov and Svergun, 2013; Schneidman-Duhovny et al., 2012). First, we applied an *ab initio* strategy to reconstruct a molecular envelope (Figure 2C) (Svergun, 1999; Svergun and Koch, 2002; Svergun et al., 2001). Second, we generated homology models for the three domains of ALC1 (Figures 2B and S2B) and optimized their positions/orientations as rigid bodies to best match the experimental scattering (Petoukhov and Svergun, 2005) (Figure 2C). Notably, both modeling approaches obtained good fits (mean $\chi^2$ = 0.95 or 1.73 for 20 independent *ab initio* shape reconstructions or rigid-body modeling trials, respectively).

All rigid-body modeling trials yielded structures with highly similar shapes. However, closer inspection revealed that the structures could be grouped into two clusters. Within each, the models exhibited extremely similar structures with near-identical translational positions and only minor differences in the rotational orientation of the three domains (Figure S2C). The defining difference between the two models is that the macro domain is positioned against the C-terminal ATPase lobe in one (ALC1$^{fl}$-MC) and against the N-terminal ATPase lobe in the other model (ALC1$^{fl}$-MN) (Figure 2C). Notably, their computed scattering profiles fit the observed data equally well ($\chi^2$ = 1.62 and $\chi^2$ = 1.72 for ALC1$^{fl}$-MC and ALC1$^{fl}$-MN, respectively). Furthermore, both ALC1$^{fl}$-MC and ALC1$^{fl}$-MN structures are in excellent agreement with *ab initio* shape reconstructions. These data suggest that the macro domain of ALC1 is in close juxtaposition with the ATPase lobes under non-activating conditions.

## Electron Microscopy Data Are Consistent with the SAXS-Based Structural Models

Next, we examined ALC1$^{fl}$ by negative stain and cryo-electron microscopy (cryo-EM) (Figures 2D and S2D). Slight blurring of features in the cryo-EM 2D-class averages indicated small-scale structural flexibility, which substantially limited resolution and precluded 3D classification and an unambiguous validation of the SAXS-based models. Nonetheless, several EM class averages (Figure 2D) exhibited three distinct areas of strong density, consistent with the arrangement of the three domains suggested by SAXS (Figures 2E and 2C). Thus, our EM analyses are consistent with both SAXS-derived structural models for ALC1$^{fl}$ but do not allow us to determine which of the two models reflects the true conformation of ALC1.

## ALC1$^{fl}$-MC and ALC1$^{fl}$-MN Co-exist in Solution

To further interrogate the inactive ALC1$^{fl}$ structure, we carried out cross-linking coupled to mass spectrometry (XL-MS) (Leitner et al., 2012; Maiolica et al., 2007; Young et al., 2000) (Figure 2F; Table S1) under conditions such that only intra- but not intermolecular cross-links could be formed (Figure S2E). Next to a number of cross-links between the two ATPase lobes, we also identified both Macro:N-ATPase and Macro:C-ATPase cross-links, suggesting that the macro domain can be found in close proximity of either ATPase lobe (Figure 2F). In light of our structural models, Macro:N-ATPase and Macro:C-ATPase cross-links appear to be mutually exclusive. Mapped onto the ALC1$^{fl}$-MC structure, all Macro:N-ATPase cross-links correspond to distances of >65 Å, substantially larger than what would be consistent with disuccinimidyl suberate (DSS)-mediated cross-linking (Merkley et al., 2014). At the same time, the ALC1$^{fl}$-MC model is entirely consistent with the N-ATPase: C-ATPase and Macro:C-ATPase cross-links. Similarly, mapping of the Macro:C-ATPase cross-links onto ALC1$^{fl}$-MN yielded implausible distances, whereas all N-ATPase:C-ATPase and Macro:N-ATPase cross-links are plausible in the context of the ALC1$^{fl}$-MN structure. Rigid-body modeling using subsets of cross-links as restraints (Petoukhov and Svergun, 2005) indicates that a model that could satisfy all three sets of cross-links at the same time is unlikely (Figure S2F). We note that observed cross-links represent the sum of cross-links that can be formed in an ensemble of several accessible conformations. Together with the SAXS and EM data, our XL-MS data therefore suggest that, in solution, ALC1 adopts two distinct co-existing conformations, ALC1$^{fl}$-MC and ALC1$^{fl}$-MN.

Consistent with this, SAXS analyses of an inactive version of ALC1 lacking the N-terminal ATPase lobe (ALC1$^{\Delta N}$) suggest that the removal of the N-terminal ATPase lobe promotes a single, compact conformation (Figure S3).

## The ALC1 ATPase Adopts an Inactive Conformation under Non-activating Conditions

To explore the structural basis for the low ATPase activity of ALC1$^{fl}$ under non-activating conditions (Figure 1A), we examined the relative orientation of the ATPase lobes (Figure 3). In either ALC1$^{fl}$-MC or ALC1$^{fl}$-MN, the relative orientation of the two ATPase lobes is similar (root-mean-square deviation [RMSD] of 5.6 Å). Strikingly, they are splayed apart to an extent that ATP hydrolysis is highly unfavorable. In contrast, in the nucleosome-bound structure of the SNF2 ATPase from *S. cerevisiae* that is believed to represent a catalytically competent state, the two lobes pack tightly against each other (Liu et al., 2017). An alignment of the ATPase of SNF2 onto that of ALC1$^{fl}$ indicates that a conversion of the ALC1$^{fl}$ ATPase into the closed conformation would necessitate a shift of the C- or N-terminal ATPase lobe by >20 Å in the case of ALC1$^{fl}$-MC or ALC1$^{fl}$-MN, respectively.

## The Binding of PARylated PARP1 to the Macro Domain Displaces It from the ATPase Motor

To examine potential interactions between the macro domain of ALC1 and its ATPase motor suggested by SAXS, EM, and XL-MS, we expressed and purified the ATPase motor (ALC1$^{cat}$) and the macro domain (ALC1$^{macro}$) as separate proteins. Remarkably, mixing them together yielded a stable complex

(E) Theoretical projections of the SAXS-based models (low-pass filtered to 15 Å) in the approximate orientations as shown in (D).
(F) Inter-domain cross-links mapped onto the ALC1$^{fl}$-MC and ALC1$^{fl}$-MN structures. Lines are colored green versus red for plausible versus non-plausible cross-links.
See also Figures S2 and S3 and Table S1.

CellPress

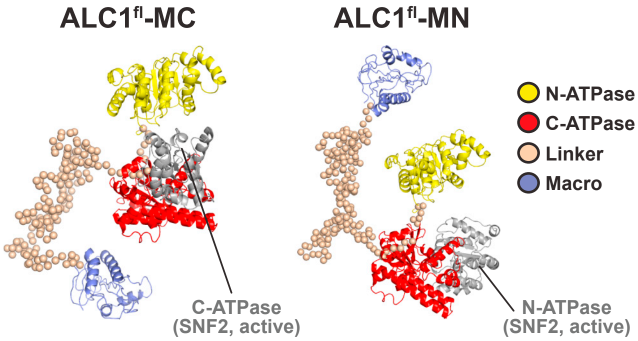


**Figure 3. The ATPase of ALC1$^{fl}$ Adopts an Inactive Conformation**

The SNF2 ATPase (Liu et al., 2017) aligned onto that of ALC1$^{fl}$-MC (left panel) or ALC1$^{fl}$-MN (right panel) using the N-lobe or the C-lobe as a guide, respectively. The ATPase lobe of SNF2 used as a guide for alignment is not shown.

(ALC1$^{complex}$) (Figures 4A, S4A, and S4B), demonstrating that the ATPase motor and macro domain of ALC1 interact in *trans*.

To determine whether this interaction is altered upon ALC1 activation, we reconstituted a complex in *trans* (ALC1$^{complex}$) from separately purified 6xHis-tagged ATPase motor (ALC1$^{cat}$) as well as non-tagged macro domain (ALC1$^{macro\Delta6xHis}$) protein (Figure 4B). Under non-activating conditions, both proteins were detected in the eluate after Ni-NTA affinity purification, further supporting the existence of an in *trans* complex. Strikingly, incubation with PARylated PARP1 significantly reduced ALC1$^{complex}$ formation as judged by the loss of ALC1$^{macro\Delta6xHis}$ from the eluate. We propose that binding of PARylated PARP1 to the macro domain displaces it from the ATPase of ALC1$^{complex}$.

To determine whether an in *trans* ALC1$^{complex}$ can be detected *in vivo*, we transfected U2OS cells with expression constructs for an YFP-tagged version of the ATPase motor (YFP-ALC1Δmacro) and a FLAG-tagged version of the macro domain (FLAG-macro). α-FLAG immunoprecipitation of the macro domain revealed an in *trans* interaction with the ATPase motor (Figures 4C and S4C) in the presence but not absence of the PARP inhibitor olaparib. Thus, the macro domain and ATPase motor of ALC1 interact under non-activating conditions *in vivo*, and these interactions are modulated by PARP activity.

## ALC1 Undergoes a Major Conformational Change upon Activation

The loss of macro domain-ATPase interaction upon incubation with PARylated PARP1 raised the possibility that a major conformational transition occurs upon activation of ALC1. To investigate this, we conducted hydrogen-deuterium exchange-mass spectrometry (HDX-MS) experiments. We first compared HDX behavior of ALC1$^{fl}$ with that of ALC1$^{fl}$ incubated with non-PARylated PARP1 (in the absence of NAD$^+$). As expected, the corresponding difference plot (Figure S5A) showed no significant differences in solvent accessibility. In stark contrast, the HDX behavior of ALC1$^{fl}$ exhibited dramatic changes upon incubation with PARylated PARP1 (Figures 5A and S5B). Solvent accessibility changed in almost all regions of the ALC1$^{fl}$; however, the most striking differences involved two regions spanning amino acid residues 328–355 and 604–639, respectively. The first region comprises acidic patch residues of the C-terminal ATPase lobe that are juxtaposed to the ALC1$^{fl}$-MC macro domain (Figure 5A) and that become substantially more exposed upon activation, consistent with a displacement of the macro domain from the ATPase motor. The second region encompasses residues of the linker proximal to the C-terminal ATPase lobe that exhibit dramatically increased protection and therefore may contribute to a PARP1-binding interface or may intimately associate with other regions of ALC1 in the context of the activated conformation.

Next, we compared intra-ALC1 cross-links upon incubation with PARP1 in the absence of NAD$^+$ (non-activated ALC1) and in its presence (activated ALC1) (Figure 5B). Although 54% of all cross-links were detected in both cases, 35% of the cross-links were not identified upon activation, and 11% were only found for the activated sample, consistent with a major PARylated PARP1-induced structural transition. The main changes involve amino acid residues 569–665, approximately matching the proximal linker that exhibited dramatically altered HDX behavior, which suggests again a role for this portion of the linker in ALC1 activation. Taken together, these data indicate that the binding of PARylated PARP1 induces a major conformational rearrangement that includes the release of the macro domain from the ATPase and a reorganization of the proximal linker.

To probe whether removal of the macro domain alone could yield the active ATPase conformation, we computed a SAXS-based rigid-body model for ALC1$^{cat}$ (Figures 5C and S5D). Interestingly, the relative positions of the two ATPase lobes were almost identical to those seen in inactive ALC1$^{fl}$ (Figure 5D). This inactive conformation of ALC1$^{cat}$ suggests that macro domain release by itself may not be sufficient to stabilize the active conformation of ALC1, in complete agreement with the low ATPase activity of ALC1$^{cat}$ (Figure 1A). Given the elevated ATPase activity of ALC1$^{cat}$ in the presence of DNA (Figure 1A) and the requirement of Snf2-family ATPase motors to adopt a closed conformation for efficient ATPase hydrolysis, we would expect the addition of DNA to induce the closure of the ALC1$^{cat}$ ATPase motor. Since SAXS analyses are sensitive to such a substantial conformational change, we repeated our measurements with ALC1$^{cat}$ in the presence of dsDNA to compare the pairwise interatomic distance distributions for ALC1$^{cat}$ in the *apo* and DNA-bound state. Indeed, the principal peak, maximum interatomic distance, and particle volume all decreased upon addition of DNA (Figure 5E). Such DNA-induced compaction is consistent with the closed configuration seen in the active conformation of Snf2-related ATPase motors (Thomä et al., 2005) and the elevated ATPase activity of ALC1$^{cat}$ in the presence but not absence of DNA (Figure 1A). We note that the inactive conformation of ALC1$^{cat}$ in the *apo* state does not imply an insignificant role of the macro domain in the regulation of the ALC1 ATPase. Rather, macro domain release appears to be a prerequisite for full activation, since in the presence of the macro domain the addition of DNA alone cannot release maximum ATPase activity of ALC1$^{fl}$ (Figure 1A).

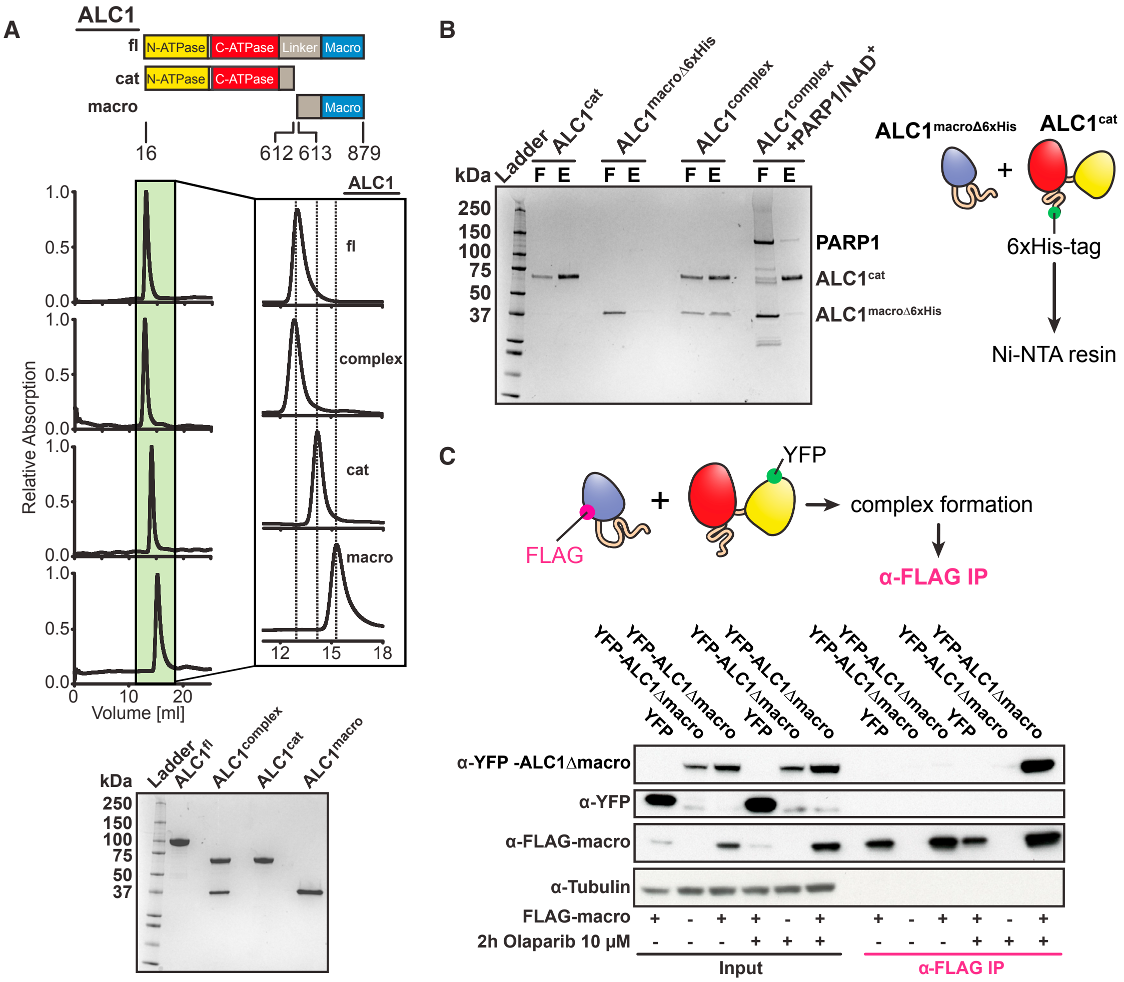


**Figure 4. The Binding of PARylated PARP1 to the Macro Domain Displaces It from the ATPase Motor**

(A) Reconstitution of an ALC1$^{cat}$:ALC1$^{macro}$ complex (ALC1$^{complex}$). Top: analytical gel filtrations. Bottom: SDS-PAGE of the peak fractions.

(B) SDS-PAGE of ALC1$^{complex}$ after Ni-NTA purification. 6xHis-tag bearing ALC1$^{cat}$ was incubated with a version of ALC1$^{macro}$ that lacks the 6xHis-tag (ALC1$^{macro\Delta6xHis}$) and purified using Ni-NTA resin.

(C) Co-immunoprecipitation (coIP) assay with plasmids expressing FLAG-tagged macro domain (FLAG-macro), YFP, or an YFP-tagged version of the ATPase (YFP-ALC1Δmacro). Cells were grown in the presence or absence of olaparib, as indicated. Cell lysates (input) were immunoprecipitated with α-FLAG. Immunoblotting was carried out with antibodies as indicated.

See also Figure S4.

## Electrostatic Interactions Play a Key Role in the Autoinhibition of ALC1$^{fl}$

In both ALC1$^{fl}$-MC and ALC1$^{fl}$-MN models the two ATPase lobes are splayed apart such that ATP hydrolysis is highly unfavorable (Figure 3). Since the ATPase motor and macro domain interaction is lost upon activation, we considered the possibility that juxtaposition of the ATPase motor and macro domain is important in maintaining ALC1 in a catalytically inactive state. We therefore sought to identify residues that are necessary for this autoinhibitory interaction. Of particular interest are the striking number of basic amino acid residues of the macro domain that are not involved in the formation of the ADP-Ribose/PAR binding pocket (Karras et al., 2005). All of these are highly conserved in ALC1/CHD1L remodelers but not in ADP-Ribose/PAR-binding macro domains from unrelated proteins (Figure S6A). In our model, these basic residues are located on helices αH3 and αH4 (Figure 6A). Intriguingly, two of these macro domain residues have been identified as sites of somatic mutation in cancer (COSMIC, catalog of somatic mutations in cancer), with cancer-associated mutations leading to a loss of the positive charge (R857Q and R860W).

Assuming a role for the macro domain in ATPase autoinhibition, we predicted R857 and and R860 to help stabilize the position of the macro domain against the ATPase. In both ALC1$^{fl}$-MC and ALC1$^{fl}$-MN, these basic residues face an acidic surface patch of the juxtaposed ATPase lobe, most likely enabling electrostatic interactions (Figure 6A). Given that SAXS as a low-resolution method cannot provide information at the atomic level about the position and orientation of individual amino acid side chains, we further examined the contributions of these basic

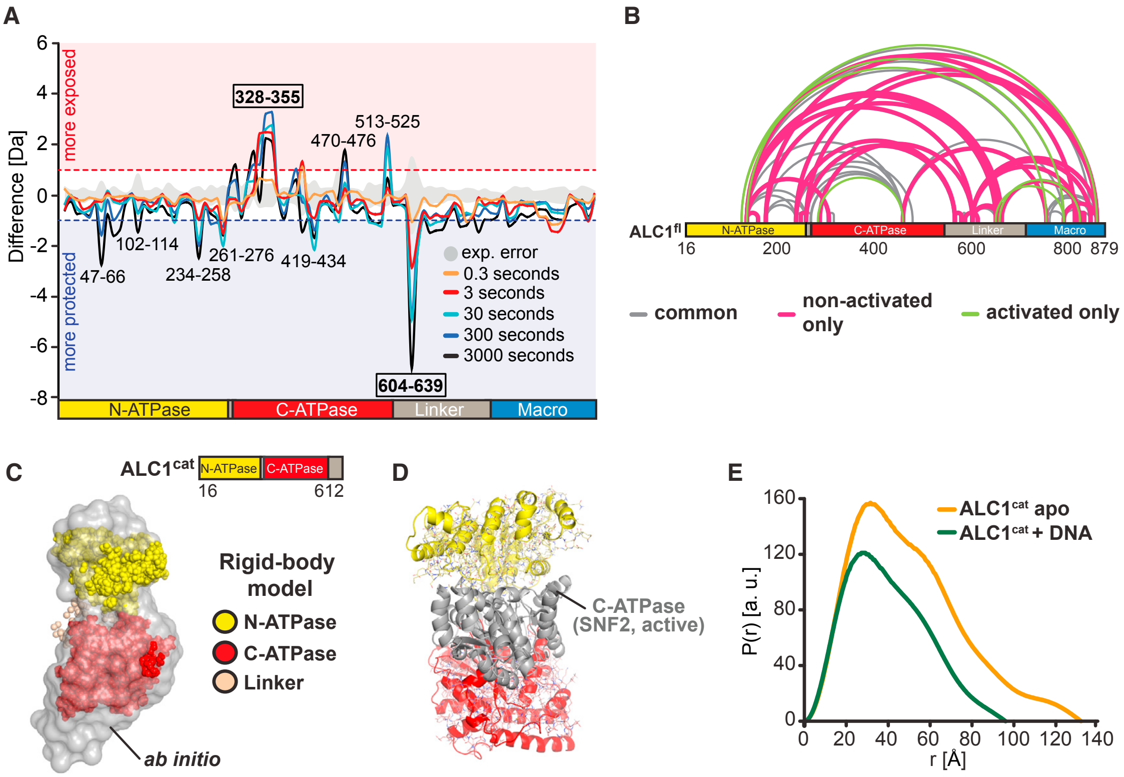


**Figure 5. The Binding of PARylated PARP1 to the Macro Domain of ALC1 Triggers a Major Conformational Change**

(A) Difference plot of HDX data from ALC1$^{fl}$ in the presence and absence of PARylated PARP1. Experimental error is shown in gray, and areas of increased exposure and protection are shaded in red and blue, respectively. Different line colors show different $D_2O$ incubation times. Non-covered sequence (Figure S5C) is omitted.

(B) Comparison of intra-ALC1 cross-links in non-activated or activated ALC1$^{fl}$. Gray, green, and magenta lines indicate cross-links that are detected in both cases, only with activated, or only with non-activated ALC1$^{fl}$, respectively.

(C) SAXS-based *ab initio* and rigid-body models of ALC1$^{cat}$.

(D) The ATPase of SNF2 (Liu et al., 2017) aligned onto that of ALC1$^{cat}$ (cartoon) and of ALC1$^{fl}$-MC (lines), using the N-lobe as a guide (not shown for SNF2).

(E) SAXS-derived pairwise interatomic distance distribution for ALC1$^{cat}$ in the *apo* (orange) and DNA-bound (green) state, scaled such that the area under the curve matches the observed particle volume.

See also Figure S5.

residues of the macro domain to interactions with the ATPase of ALC1 *in vivo*. We transfected U2OS cells with expression constructs for wild-type (WT) or mutant FLAG-tagged versions of the macro domain (FLAG-macro) as well as an YFP-tagged version of the ATPase, YFP-ALC1Δmacro (Figures 6B and S6B). Upon α-FLAG immunoprecipitation and in the presence of olaparib, a substantial amount of the ATPase motor precipitated with WT but not with R860W, R857Q, or R857E FLAG-macro. We also carried out analogous experiments with a FLAG-tagged version of the WT macro domain as well as an YFP-tagged version of intact ALC1 bearing macro domain mutations. If such mutations were to destabilize macro domain-ATPase motor interactions, we would expect separately expressed FLAG-macro to more easily compete with the mutated macro domain of intact ALC1 for interactions with the ATPase motor. Indeed, upon α-FLAG immunoprecipitation and in the presence of olaparib, substantial amounts of intact ALC1 precipitated with FLAG-macro only for the R860W, R857Q, or R857E mutated versions of intact ALC1 but not for WT ALC1 (Figures 6C and S6C). Taken together, our experiments indicate that these mutations destabilize interactions between the macro and ATPase domains of ALC1.

In both ALC1$^{fl}$-MC and ALC1$^{fl}$-MN, the basic patch of the macro domain is juxtaposed to an acidic surface patch of the respective ATPase lobe (Figure 6A), which features conserved negatively charged residues (Figure S6A). To examine their contributions to the ATPase motor-macro domain interaction, we repeated the interaction assay with YFP-tagged versions of the ATPase motor bearing acidic patch mutations on either the C-terminal or N-terminal ATPase lobe (Figures 6D and 6E, respectively; see also Figures S6D and S6E). Strikingly, several mutations, including the cancer-associated mutation D340Y, destabilized interactions of the macro domain with the C-terminal ATPase lobe. In contrast, none of the acidic patch mutations of the N-terminal ATPase lobe or its deletion altogether (Figures 6E and S6E) had any appreciable impact on ATPase motor-macro domain interactions. Taken together, these data suggest that *in vivo*, stable interactions between the macro

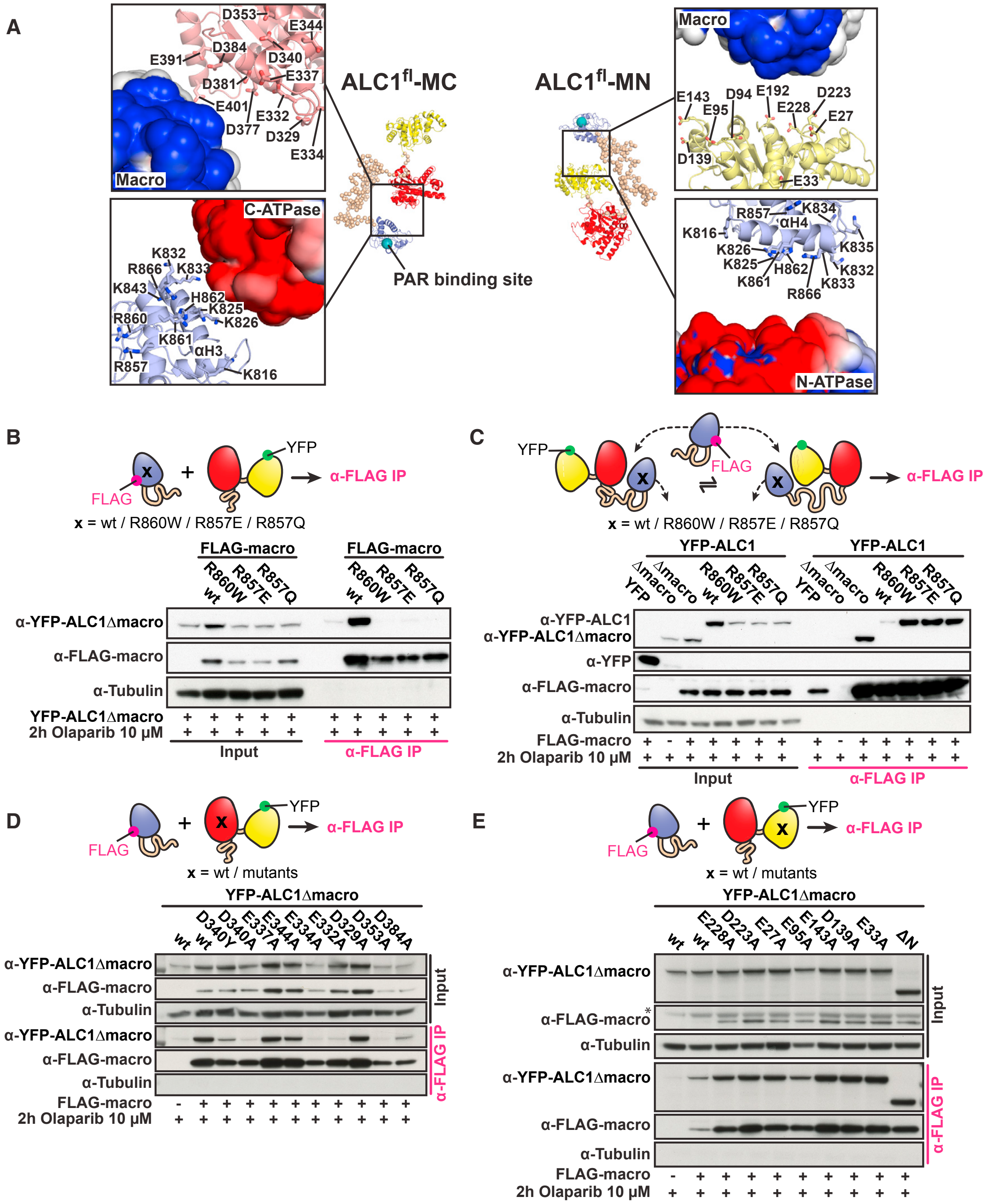


**Figure 6. Conserved Electrostatic Interactions Stabilize the Juxtaposition of the Macro Domain against Predominantly the C-Terminal ATPase Lobe**

(A) Macro domain placement against the juxtaposed ATPase lobe. Surfaces are colored on a red-white-blue gradient from −2 kT/e to +2 kT/e, as calculated by Adaptive Poisson-Boltzmann Solver (APBS) (Baker et al., 2001; Dolinsky et al., 2007). Cyan sphere: PAR-binding site, from an alignment with the Af1521 macro domain (Karras et al., 2005).

(B–E) CoIP assays to probe macro domain-ATPase motor interactions. As indicated, cells were transfected with plasmids expressing mutated (B) or WT (C) FLAG-tagged macro domain (FLAG-macro), YFP-tagged ATPase motor (YFP-ALC1Δmacro) bearing mutations on the C-terminal (D) or N-terminal (E) ATPase lobe, YFP-tagged ALC1 lacking both macro domain and the N-terminal ATPase lobe (YFP-ALC1ΔNΔmacro) (E), and/or YFP-tagged intact ALC1 bearing macro domain mutations (C). Cells were grown in the presence or absence of olaparib, as indicated. Cell lysates were immunoprecipitated with α-FLAG. The cell lysates (input) and immunoprecipitates were analyzed by immunoblotting with the indicated antibodies. The asterisk denotes a non-specific band.

See also Figure S6.

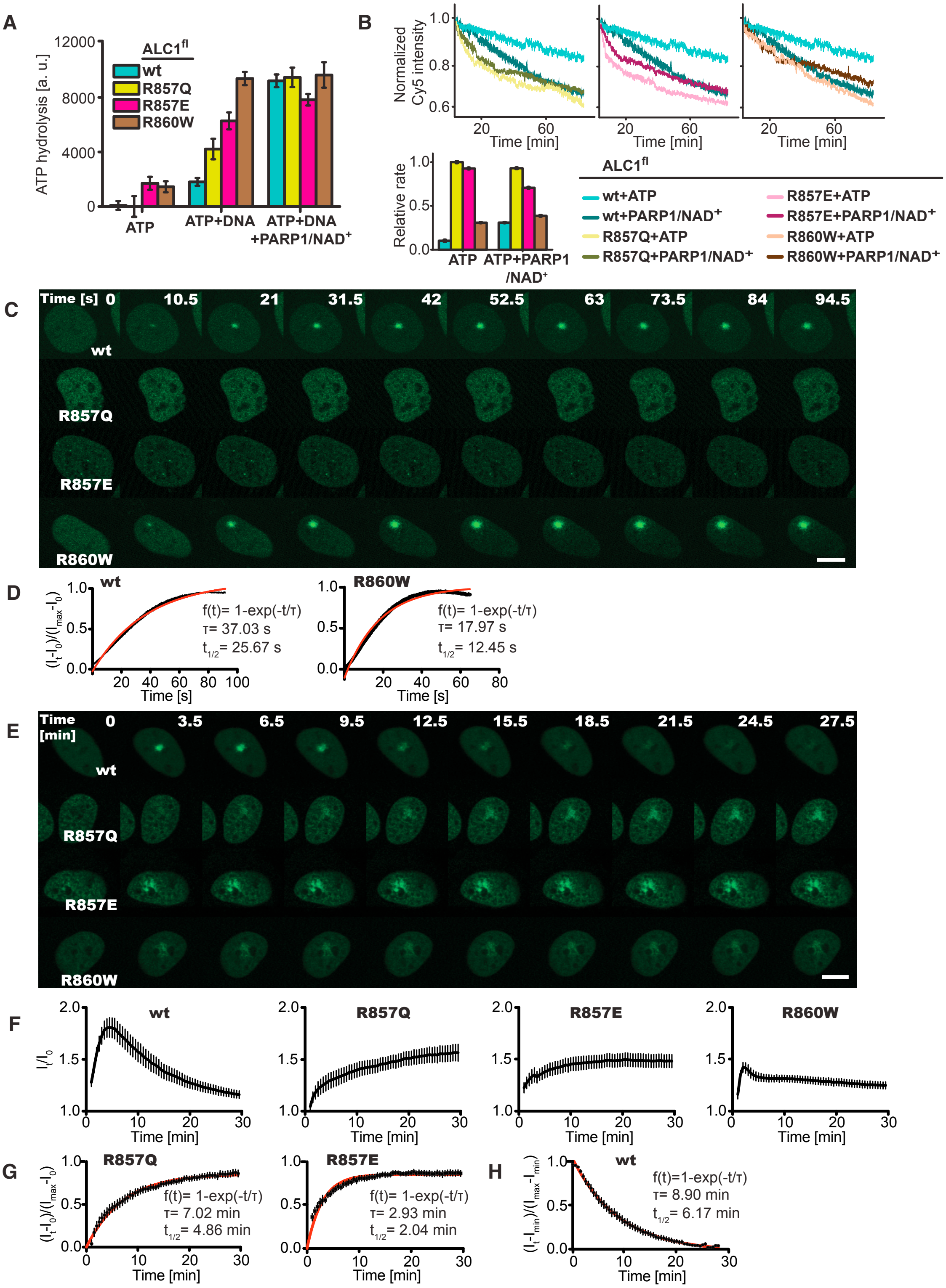


**Figure 7. Mutations that Destabilize ATPase Motor-Macro Domain Interactions Constitutively Activate ALC1 and Alter the Dynamics of Its Recruitment at DNA Damage Sites**

(A) ATPase activities of ALC1$^{fl}$ WT (teal), R857Q (green), R857E (magenta), and R860W (brown).

(B) Remodeling by ALC1$^{fl}$ WT (same as in Figure 1C) compared to R857Q (left), R857E (middle), and R860W (right) in the presence or absence of PARP1/NAD$^+$. Bar graph: relative rates for ALC1$^{fl}$ WT (teal), R857Q (green), R857E (magenta), and R860W (brown).

(legend continued on next page)

and ATPase motor domains of ALC1 require the acidic patch of the C-terminal but not the N-terminal ATPase lobe.

To examine the effect of the ATPase motor-macro domain interaction on the regulation of ALC1, we purified ALC1$^{fl}$ bearing the somatic cancer mutations R860W or R857Q, or the mutation R857E. In the presence of DNA, all three mutants exhibited elevated ATPase activity compared to WT ALC1$^{fl}$ (Figure 7A). Most strikingly, the addition of DNA augmented the ATPase activity of ALC1$^{fl}$ R860W by ~6.5-fold to approximately the same activity as that observed with PARP1/NAD$^+$ for WT ALC1$^{fl}$ or ALC1$^{fl}$ R860W. Similarly, when compared to WT ALC1$^{fl}$, all three mutants displayed markedly increased remodeling rates without PARP1/NAD$^+$ (3.0-, 9.8-, and 9.1-fold for ALC1$^{fl}$ R860W, R857Q, and R857E, respectively) (Figure 7B). For ALC1$^{fl}$ R857Q or R857E, these increased rates in the absence of PARP1/NAD$^+$ were larger (by a factor of 3.2 or 3.0, respectively) than the remodeling rate observed for WT ALC1$^{fl}$ with PARP1/NAD$^+$.

To examine the impact of destabilizing the interaction between the macro domain and ATPase motor of ALC1 on its function *in vivo*, we engineered R860W, R857Q, and R857E mutations into an YFP-ALC1 construct to monitor recruitment kinetics at sites of DNA damage (Ahel et al., 2009). Strikingly, both R857Q and R857E mutations compromised the recruitment of ALC1 to damage sites at early time points, which contrasted with the rapid accumulation of WT and the R860W protein (Figures 7C and 7D). ALC1 R857Q and R857E mutations eventually accumulate at damage sites, but with delayed kinetics relative to the WT (Figures 7E–7G). Given the proximity of the R857 residue to the PAR-binding site within the macro domain, it is likely that the R857Q and R857E mutations impact on PAR binding, which is required for optimal recruitment to damage sites. Indeed, ALC1 R857E exhibited moderately reduced PAR binding *in vitro* (Figure S7). Hence, although the ALC1 R857Q and R857E mutants are constitutively activated *in vitro*, their ability to recognize and bind PAR chains at damage sites *in vivo* is impaired.

Interestingly, the R860W mutant accumulates more rapidly than the WT protein (Figure 7G). A proportion of the R860W mutant molecules rapidly dissociates from sites of damage immediately after maximal recruitment. However, following this initial dissociation, the R860W mutant persists at damage sites for longer than the WT protein (Figures 7E, 7F, and 7H). Thus, interfering with the autoinhibitory region of ALC1 affects both the association and disassociation kinetics of ALC1 at damage sites.

## DISCUSSION

Our work has uncovered an unappreciated functional crosstalk between the PAR-binding macro domain and the ATPase motor, which is important for autoinhibition of ALC1 ATPase in the inactive state.

An autoinhibitory activity of the macro domain was suggested by the observation that removal of the macro domain of ALC1 constitutively activated the ATPase and abolished its dependence upon activation by PARP1/NAD$^+$. Second, macro domain removal led to an almost complete loss of nucleosome remodeling despite elevated basal ATPase activity, suggesting a requirement of the macro domain for efficient coupling of ATP hydrolysis to nucleosome mobilization. Indeed, we found that the macro domain of ALC1 can bind nucleosomes and linker DNA. The macro domain thus may, once released from its autoinhibitory interactions with the ATPase, serve as an anchor for the conversion of ATPase-induced DNA translocation into nucleosome movement.

Given its autoinhibitory role, it appeared likely that the macro domain of ALC1 interacts directly with the ATPase to regulate its activity. Indeed, we demonstrate interactions between separately expressed ATPase motor and macro domain portions of ALC1 *in vitro* and *in vivo*. In solution, ALC1 can adopt two distinct "off" conformations that differ in the position of the macro domain either against the C-terminal or the N-terminal ATPase lobe. Irrespective of conformation, the two ATPase lobes are found splayed apart. This catalytically inactive conformation is likely maintained by the position of the macro domain against the juxtaposed ATPase lobe.

We discovered a set of conserved basic residues of the macro domain that are largely distinct from the PAR-binding pocket and appear to engage in electrostatic interactions with a negatively charged patch on the juxtaposed ATPase lobe. Our data show that cancer-associated mutations at these sites involving a loss of positive charge destabilize autoinhibitory interactions between the macro domain and the ATPase motor of ALC1 and yield elevated ATPase and nucleosome remodeling activities independent of activation by PARP1/NAD$^+$.

*In vitro*, the presence of the two distinct conformations of autoinhibited ALC1 is supported by our SAXS-based and XL-MS analyses. However, our data do not allow us to predict the relative contributions of the two conformations. Thus, one of the two conformations could be rarely populated in solution and/or represent a less important conformational state of ALC1 *in vivo*. Indeed, our cell-based assays revealed that several residues of the C-terminal ATPase acidic patch, including a site of somatic mutation in cancer, are required for stable interactions of the ATPase motor with the macro domain. In contrast, neither acidic patch mutation of the N-terminal ATPase lobe nor its deletion altogether appreciably altered ATPase motor-macro domain interactions *in vivo*. Moreover, we found residues within the acidic

(C) Representative images of U2OS cells expressing WT, R857Q, R857E, and R860W YFP-ALC1, taken at the indicated early time points following laser damage.
(D) Kinetics of YFP-ALC1 (left) and YFP-ALC1 R860W (right) association with DNA breaks as quantified from (C).
(E) Half-hour time course with representative images of U2OS cells expressing WT, R857Q, R857E, and R860W YFP-ALC1, taken at indicated time points following laser damage.
(F) Kinetics of WT, R857Q, R857E, and R860W YFP-ALC1 association with/dissociation from DNA breaks as quantified from (E).
(G) Kinetics of YFP-ALC1 R857Q and R857E association with DNA breaks.
(H) Kinetics of WT YFP-ALC1 dissociation from DNA breaks.
Error bars ± SEM; $\tau$: time constant; scale bars, 10 $\mu$m. See also Figure S7.

patch of the C-terminal but not the N-terminal ATPase lobe to become substantially more exposed upon activation. Our data therefore strongly indicate that ALC1$^{fl}$-MC may be the "off" conformation most relevant for the regulation of ALC1 catalytic activity in cells.

Live-cell imaging of macro domain mutants binding to sites of DNA damage established that preventing autoinhibition can lead to more rapid recruitment of ALC1 to damage sites, but at a cost. Preventing autoinhibition can also uncouple ATP hydrolysis and nucleosome remodeling, which is required for the ordered disassembly of ALC1 from damage sites (Ahel et al., 2009). As such, loss of autoinhibition can lead to abnormal dissociation rates due to uncontrolled ATP hydrolysis.

Our *in vitro* reconstitution, cell-based, HDX-MS, and XL-MS experiments together show that the binding of PARylated PARP1 to ALC1 triggers a major conformational change that releases interactions between the macro domain of ALC1 with its ATPase motor. How could engagement of the macro domain of ALC1 displace it from its ATPase motor? The ALC1 macro domain has negligible affinity for monomeric ADP-ribose, and foot-printing estimated that within an activated complex containing PARylated PARP1 and ALC1, 3-20 ADP-ribose units are protected from digestion (Gottschalk et al., 2012). The location of the PAR-binding site away from the ATPase (Figure 6A) does not suggest an obvious mechanism by which binding of PARylated PARP1 to the macro domain might activate the ATPase of ALC1. We note that additional subunits of the highly negatively charged PAR polymer extending out from the ADP-ribose binding site or a negatively charged patch on the surface of PARP1 could compete with the acidic ATPase motor surface for binding to the basic patch of the macro domain and thereby release the autoinhibitory interaction between the ATPase and the macro domain. Furthermore, we observed that the proximal linker region becomes protected in the presence of PARylated PARP1, suggesting a possible role of this region in an activated ALC1 conformation.

The release of the macro domain from the autoinhibited ATPase of ALC1 is a prerequisite for activation, since even in the presence of DNA, ALC1$^{fl}$ exhibits very low basal ATPase activity (Figure 1A). However, macro domain release by itself is likely not sufficient to fully stabilize the catalytically competent ATPase conformation. Indeed, we found that the isolated ATPase adopts an inactive and open conformation until bound by DNA, consistent with the low basal ATPase activity of ALC1$^{cat}$ (Figure 1A).

Taken together, our data are consistent with the following model for the regulation of ALC1 by its macro domain: association of the macro domain of ALC1 predominantly with its C-terminal ATPase lobe stabilizes an open and catalytically inactive ATPase conformation. This mode of autoinhibition would prevent undesired ATP hydrolysis without accumulation of PARylated PARP1 at sites of DNA damage. Selective activation of ALC1 occurs as a two-step process. First, the binding of PARylated PARP1 to the macro domain displaces it from the ATPase. In a second step, nucleosome/DNA binding as well as additional interactions involving the proximal linker region of ALC1 stabilize the catalytically competent, closed conformation of the ATPase. Once released from the autoinhibited conformation, the macro domain serves as an anchor required to efficiently couple ATPase-induced DNA translocation to nucleosome mobilization.

Snf2 proteins share a highly conserved ATPase that can be subject to sophisticated regulation in adaptation to a wide range of functions (Narlikar et al., 2013). In the case of the remodelers, such adaptation is in part due to the recruitment to specific features of chromatin via accessory domains. However, in addition to their role in recruitment, accessory domains can also directly regulate the catalytic activity of the ATPase domain (Narlikar et al., 2013). The macro domain, which is known to play a role in the specific recruitment of ALC1 to PARylated sites of DNA damage, is now revealed to directly impinge on the catalytic activity of the ATPase.

## STAR★METHODS

Detailed methods are provided in the online version of this paper and include the following:

- KEY RESOURCES TABLE
- CONTACT FOR REAGENT AND RESOURCE SHARING
- EXPERIMENTAL MODEL AND SUBJECT DETAILS
- METHOD DETAILS
  - Cloning, Plasmids, and Cell Lines
  - Protein Production and Purification
  - Dynamic Light Scattering (DLS)
  - PAR Binding Assay
  - ATPase Activity Measurements
  - Restriction Enzyme Accessibility Assay for Nucleosome Remodeling
  - FRET Assay for Nucleosome Remodeling
  - Reconstitution of ALC1$^{complex}$
  - Electrophoretic Mobility Shift Assay (EMSA)
  - Homology Modeling
  - Molecular Dynamics Simulations
  - Small Angle X-Ray Scattering (SAXS)
  - Sample Preparation for Electron Microscopy, Data Collection, and Analysis
  - Cross-Linking Coupled to Mass Spectrometry (XL-MS)
  - Purification of In *Trans* Complex over Ni-NTA Resin
  - Immunoprecipitation (IP)
  - Hydrogen-Deuterium Exchange Mass Spectrometry (HDX-MS)
  - Laser Micro-irradiation Induced DNA Damage
- QUANTIFICATION AND STATISTICAL ANALYSIS
- DATA AND SOFTWARE AVAILABILITY

## SUPPLEMENTAL INFORMATION

Supplemental Information includes seven figures and two tables and can be found with this article online at https://doi.org/10.1016/j.molcel.2017.10.017.

## AUTHOR CONTRIBUTIONS

S.D. conceived of and oversaw the project; L.C.L. purified proteins and carried out and analyzed activity, SAXS, and DLS measurements; G.H. and S.J.B. designed, performed, and analyzed all cell-based experiments; S.A. performed EM and analyzed the data; A.L. carried out XL-MS analyses; S.L.M. and

J.M.S. carried out HDX-MS analyses; E.M. and D.v.d.S. performed molecular dynamics (MD) simulations; V.M. and A.M. carried out PAR binding experiments; Y.C. purified proteins; S.D. and S.J.B. wrote the paper; all authors discussed and edited the manuscript.

## ACKNOWLEDGMENTS

We thank Evalena Andersson for initial work; Greg Bowman for nucleosomes; Stefan Knight, Markus Seeliger, and Sofia Pytharopoulou for discussions; Ruedi Aebersold for access to instrumentation; Robert Rambo, Nathan Cowieson, and the staff at DLS and ESRF for generous technical support. S.D. acknowledges support from a European Research Council (ERC) Starting Grant (ChromatinRemodelling), the Swedish Research Council (VR 2015-04568), the Knut and Alice Wallenberg Foundation (KAW/WAF 2014.0183), and the SciLifeLab. A.L. received funding from the ETH Zurich. The Boulton Lab is supported by the Francis Crick Institute, which receives its core funding from Cancer Research UK (FC0010048), the UK Medical Research Council (FC0010048), and the Wellcome Trust (FC0010048). S.J.B. is also funded by an ERC Advanced Investigator (TelMetab) and a Wellcome Trust Senior Investigator Grant. D.v.d.S. acknowledges a VR computer time grant (SNIC2013-26-6) and S.A. a FEBS Long-Term Fellowship.

Received: May 7, 2017
Revised: August 16, 2017
Accepted: October 17, 2017
Published: December 7, 2017

## REFERENCES

Ahel, D., Horejsí, Z., Wiechens, N., Polo, S.E., Garcia-Wilson, E., Ahel, I., Flynn, H., Skehel, M., West, S.C., Jackson, S.P., et al. (2009). Poly(ADP-ribose)-dependent regulation of DNA repair by the chromatin remodeling enzyme ALC1. Science *325*, 1240–1243.

Baker, N.A., Sept, D., Joseph, S., Holst, M.J., and McCammon, J.A. (2001). Electrostatics of nanosystems: Application to microtubules and the ribosome. Proc. Natl. Acad. Sci. USA *98*, 10037–10041.

Bartholomew, B. (2014). Regulating the chromatin landscape: Structural and mechanistic perspectives. Annu. Rev. Biochem. *83*, 671–696.

Becker, P.B., and Workman, J.L. (2013). Nucleosome remodeling and epigenetics. Cold Spring Harb. Perspect. Biol. *5*. Published online September 1, 2013. https://doi.org/10.1101/cshperspect.a017905.

Berendsen, H.J.C., Vanderspoel, D., and Vandrunen, R. (1995). Gromacs—a message-passing parallel molecular-dynamics implementation. Comput. Phys. Commun. *91*, 43–56.

Bowman, G.D. (2010). Mechanisms of ATP-dependent nucleosome sliding. Curr. Opin. Struct. Biol. *20*, 73–81.

Brune, M., Hunter, J.L., Corrie, J.E., and Webb, M.R. (1994). Direct, real-time measurement of rapid inorganic phosphate release using a novel fluorescent probe and its application to actomyosin subfragment 1 ATPase. Biochemistry *33*, 8262–8271.

Chen, M., Huang, J.D., Hu, L., Zheng, B.J., Chen, L., Tsang, S.L., and Guan, X.Y. (2009). Transgenic CHD1L expression in mouse induces spontaneous tumors. PLoS ONE *4*, e6727.

Chen, L., Chan, T.H., Yuan, Y.F., Hu, L., Huang, J., Ma, S., Wang, J., Dong, S.S., Tang, K.H., Xie, D., et al. (2010). CHD1L promotes hepatocellular carcinoma progression and metastasis in mice and is associated with these processes in human patients. J. Clin. Invest. *120*, 1178–1191.

Clapier, C.R., and Cairns, B.R. (2009). The biology of chromatin remodeling complexes. Annu. Rev. Biochem. *78*, 273–304.

D'Amours, D., Desnoyers, S., D'Silva, I., and Poirier, G.G. (1999). Poly(ADP-ribosyl)ation reactions in the regulation of nuclear functions. Biochem. J. *342*, 249–268.

de Murcia, G., Huletsky, A., Lamarre, D., Gaudreau, A., Pouyet, J., Daune, M., and Poirier, G.G. (1986). Modulation of chromatin superstructure induced by poly(ADP-ribose) synthesis and degradation. J. Biol. Chem. *261*, 7011–7017.

Dolinsky, T.J., Czodrowski, P., Li, H., Nielsen, J.E., Jensen, J.H., Klebe, G., and Baker, N.A. (2007). PDB2PQR: Expanding and upgrading automated preparation of biomolecular structures for molecular simulations. Nucleic Acids Res. *35*, W522–W525.

Dürr, H., Körner, C., Müller, M., Hickmann, V., and Hopfner, K.P. (2005). X-ray structures of the Sulfolobus solfataricus SWI2/SNF2 ATPase core and its complex with DNA. Cell *121*, 363–373.

El-Khamisy, S.F., Masutani, M., Suzuki, H., and Caldecott, K.W. (2003). A requirement for PARP-1 for the assembly or stability of XRCC1 nuclear foci at sites of oxidative DNA damage. Nucleic Acids Res. *31*, 5526–5533.

Ferreira, H., Flaus, A., and Owen-Hughes, T. (2007). Histone modifications influence the action of Snf2 family remodelling enzymes by different mechanisms. J. Mol. Biol. *374*, 563–579.

Flaus, A., Martin, D.M., Barton, G.J., and Owen-Hughes, T. (2006). Identification of multiple distinct Snf2 subfamilies with conserved structural motifs. Nucleic Acids Res. *34*, 2887–2905.

Frechette, A., Huletsky, A., Aubin, R.J., de Murcia, G., Mandel, P., Lord, A., Grondin, G., and Poirier, G.G. (1985). Poly(ADP-ribosyl)ation of chromatin: Kinetics of relaxation and its effect on chromatin solubility. Can. J. Biochem. Cell Biol. *63*, 764–773.

Gottschalk, A.J., Timinszky, G., Kong, S.E., Jin, J., Cai, Y., Swanson, S.K., Washburn, M.P., Florens, L., Ladurner, A.G., Conaway, J.W., and Conaway, R.C. (2009). Poly(ADP-ribosyl)ation directs recruitment and activation of an ATP-dependent chromatin remodeler. Proc. Natl. Acad. Sci. USA *106*, 13770–13774.

Gottschalk, A.J., Trivedi, R.D., Conaway, J.W., and Conaway, R.C. (2012). Activation of the SNF2 family ATPase ALC1 by poly(ADP-ribose) in a stable ALC1·PARP1·nucleosome intermediate. J. Biol. Chem. *287*, 43527–43532.

Hassa, P.O., Haenni, S.S., Elser, M., and Hottiger, M.O. (2006). Nuclear ADP-ribosylation reactions in mammalian cells: Where are we today and where are we going? Microbiol. Mol. Biol. Rev. *70*, 789–829.

Hauk, G., and Bowman, G.D. (2011). Structural insights into regulation and action of SWI2/SNF2 ATPases. Curr. Opin. Struct. Biol. *21*, 719–727.

Hauk, G., McKnight, J.N., Nodelman, I.M., and Bowman, G.D. (2010). The chromodomains of the Chd1 chromatin remodeler regulate DNA access to the ATPase motor. Mol. Cell *39*, 711–723.

Hopfner, K.P., Gerhold, C.B., Lakomek, K., and Wollmann, P. (2012). Swi2/Snf2 remodelers: Hybrid views on hybrid molecular machines. Curr. Opin. Struct. Biol. *22*, 225–233.

Hura, G.L., Menon, A.L., Hammel, M., Rambo, R.P., Poole, F.L., 2nd, Tsutakawa, S.E., Jenney, F.E., Jr., Classen, S., Frankel, K.A., Hopkins, R.C., et al. (2009). Robust, high-throughput solution structural analyses by small angle X-ray scattering (SAXS). Nat. Methods *6*, 606–612.

Karras, G.I., Kustatscher, G., Buhecha, H.R., Allen, M.D., Pugieux, C., Sait, F., Bycroft, M., and Ladurner, A.G. (2005). The macro domain is an ADP-ribose binding module. EMBO J. *24*, 1911–1920.

Leduc, Y., de Murcia, G., Lamarre, D., and Poirier, G.G. (1986). Visualization of poly(ADP-ribose) synthetase associated with polynucleosomes by immunoelectron microscopy. Biochim. Biophys. Acta *885*, 248–255.

Leitner, A., Reischl, R., Walzthoeni, T., Herzog, F., Bohn, S., Forster, F., and Aebersold, R. (2012). Expanding the chemical cross-linking toolbox by the use of multiple proteases and enrichment by size exclusion chromatography. Mol. Cell. Proteomics *11*. M111.014126. Published online March 1, 2012. https://doi.org/10.1074/mcp.M111.014126.

Li, X., Mooney, P., Zheng, S., Booth, C.R., Braunfeld, M.B., Gubbens, S., Agard, D.A., and Cheng, Y. (2013). Electron counting and beam-induced motion correction enable near-atomic-resolution single-particle cryo-EM. Nat. Methods *10*, 584–590.

Lindahl, T., Satoh, M.S., and Dianov, G. (1995). Enzymes acting at strand interruptions in DNA. Philos. Trans. R. Soc. Lond. B Biol. Sci. *347*, 57–62.

Liu, X., Li, M., Xia, X., Li, X., and Chen, Z. (2017). Mechanism of chromatin remodelling revealed by the Snf2-nucleosome structure. Nature *544*, 440–445.

Ma, N.F., Hu, L., Fung, J.M., Xie, D., Zheng, B.J., Chen, L., Tang, D.J., Fu, L., Wu, Z., Chen, M., et al. (2008). Isolation and characterization of a novel oncogene, amplified in liver cancer 1, within a commonly amplified region at 1q21 in hepatocellular carcinoma. Hepatology *47*, 503–510.

Maiolica, A., Cittaro, D., Borsotti, D., Sennels, L., Ciferri, C., Tarricone, C., Musacchio, A., and Rappsilber, J. (2007). Structural analysis of multiprotein complexes by cross-linking, mass spectrometry, and database searching. Mol. Cell. Proteomics *6*, 2200–2211.

Malanga, M., and Althaus, F.R. (2005). The role of poly(ADP-ribose) in the DNA damage signaling network. Biochem. Cell Biol. *83*, 354–364.

Marchio, A., Meddeb, M., Pineau, P., Danglot, G., Tiollais, P., Bernheim, A., and Dejean, A. (1997). Recurrent chromosomal abnormalities in hepatocellular carcinoma detected by comparative genomic hybridization. Genes Chromosomes Cancer *18*, 59–65.

Merkley, E.D., Rysavy, S., Kahraman, A., Hafen, R.P., Daggett, V., and Adkins, J.N. (2014). Distance restraints from crosslinking mass spectrometry: mining a molecular dynamics simulation database to evaluate lysine-lysine distances. Protein Sci. *23*, 747–759.

Mevissen, T.E.T., Kulathu, Y., Mulder, M.P.C., Geurink, P.P., Maslen, S.L., Gersch, M., Elliott, P.R., Burke, J.E., van Tol, B.D.M., Akutsu, M., et al. (2016). Molecular basis of Lys11-polyubiquitin specificity in the deubiquitinase Cezanne. Nature *538*, 402–405.

Mu, Q.J., Li, H.L., Yao, Y., Liu, S.C., Yin, C.G., and Ma, X.Z. (2015). Chromodomain helicase/ATPase DNA-binding protein 1-like gene (CHD1L) expression and implications for invasion and metastasis of breast cancer. PLoS ONE *10*, e0143030.

Narlikar, G.J., Sundaramoorthy, R., and Owen-Hughes, T. (2013). Mechanisms and functions of ATP-dependent chromatin-remodeling enzymes. Cell *154*, 490–503.

Petoukhov, M.V., and Svergun, D.I. (2005). Global rigid body modeling of macromolecular complexes against small-angle scattering data. Biophys. J. *89*, 1237–1250.

Petoukhov, M.V., and Svergun, D.I. (2013). Applications of small-angle X-ray scattering to biomacromolecular solutions. Int. J. Biochem. Cell Biol. *45*, 429–437.

Petoukhov, M.V., Franke, D., Shkumatov, A.V., Tria, G., Kikhney, A.G., Gajda, M., Gorba, C., Mertens, H.D., Konarev, P.V., and Svergun, D.I. (2012). New developments in the ATSAS program package for small-angle scattering data analysis. J. Appl. Cryst. *45*, 342–350.

Pines, A., Vrouwe, M.G., Marteijn, J.A., Typas, D., Luijsterburg, M.S., Cansoy, M., Hensbergen, P., Deelder, A., de Groot, A., Matsumoto, S., et al. (2012). PARP1 promotes nucleotide excision repair through DDB2 stabilization and recruitment of ALC1. J. Cell Biol. *199*, 235–249.

Poirier, G.G., de Murcia, G., Jongstra-Bilen, J., Niedergang, C., and Mandel, P. (1982). Poly(ADP-ribosyl)ation of polynucleosomes causes relaxation of chromatin structure. Proc. Natl. Acad. Sci. USA *79*, 3423–3427.

Realini, C.A., and Althaus, F.R. (1992). Histone shuttling by poly(ADP-ribosylation). J. Biol. Chem. *267*, 18858–18865.

Satoh, M.S., and Lindahl, T. (1992). Role of poly(ADP-ribose) formation in DNA repair. Nature *356*, 356–358.

Scheres, S.H. (2012). RELION: implementation of a Bayesian approach to cryo-EM structure determination. J. Struct. Biol. *180*, 519–530.

Scheres, S.H. (2015). Semi-automated selection of cryo-EM particles in RELION-1.3. J. Struct. Biol. *189*, 114–122.

Schneidman-Duhovny, D., Kim, S.J., and Sali, A. (2012). Integrative structural modeling with small angle X-ray scattering profiles. BMC Struct. Biol. *12*, 17.

Sellou, H., Lebeaupin, T., Chapuis, C., Smith, R., Hegele, A., Singh, H.R., Kozlowski, M., Bultmann, S., Ladurner, A.G., Timinszky, G., and Huet, S. (2016). The poly(ADP-ribose)-dependent chromatin remodeler Alc1 induces local chromatin relaxation upon DNA damage. Mol. Biol. Cell *27*, 3791–3799.

Silva, J.C., Denny, R., Dorschel, C.A., Gorenstein, M., Kass, I.J., Li, G.Z., McKenna, T., Nold, M.J., Richardson, K., Young, P., and Geromanos, S. (2005). Quantitative proteomic analysis by accurate mass retention time pairs. Anal. Chem. *77*, 2187–2200.

Singleton, M.R., Dillingham, M.S., and Wigley, D.B. (2007). Structure and mechanism of helicases and nucleic acid translocases. Annu. Rev. Biochem. *76*, 23–50.

Svergun, D.I. (1999). Restoring low resolution structure of biological macromolecules from solution scattering using simulated annealing. Biophys. J. *76*, 2879–2886.

Svergun, D.I., and Koch, M.H. (2002). Advances in structure analysis using small-angle scattering in solution. Curr. Opin. Struct. Biol. *12*, 654–660.

Svergun, D.I., Petoukhov, M.V., and Koch, M.H. (2001). Determination of domain structure of proteins from X-ray solution scattering. Biophys. J. *80*, 2946–2953.

Thomä, N.H., Czyzewski, B.K., Alexeev, A.A., Mazin, A.V., Kowalczykowski, S.C., and Pavletich, N.P. (2005). Structure of the SWI2/SNF2 chromatin-remodeling domain of eukaryotic Rad54. Nat. Struct. Mol. Biol. *12*, 350–356.

Van der Spoel, D., Lindahl, E., Hess, B., Groenhof, G., Mark, A.E., and Berendsen, H.J.C. (2005). GROMACS: fast, flexible, and free. J. Comput. Chem. *26*, 1701–1718.

Walzthoeni, T., Claassen, M., Leitner, A., Herzog, F., Bohn, S., Förster, F., Beck, M., and Aebersold, R. (2012). False discovery rate estimation for cross-linked peptides identified by mass spectrometry. Nat. Methods *9*, 901–903.

Wong, N., Chan, A., Lee, S.W., Lam, E., To, K.F., Lai, P.B., Li, X.N., Liew, C.T., and Johnson, P.J. (2003). Positional mapping for amplified DNA sequences on 1q21-q22 in hepatocellular carcinoma indicates candidate genes overexpression. J. Hepatol. *38*, 298–306.

Xia, X., Liu, X., Li, T., Fang, X., and Chen, Z. (2016). Structure of chromatin remodeler Swi2/Snf2 in the resting state. Nat. Struct. Mol. Biol. *23*, 722–729.

Yan, L., Wang, L., Tian, Y., Xia, X., and Chen, Z. (2016). Structure and regulation of the chromatin remodeller ISWI. Nature *540*, 466–469.

Yang, J.G., Madrid, T.S., Sevastopoulos, E., and Narlikar, G.J. (2006). The chromatin-remodeling enzyme ACF is an ATP-dependent DNA length sensor that regulates nucleosome spacing. Nat. Struct. Mol. Biol. *13*, 1078–1083.

Yang, J., Yan, R., Roy, A., Xu, D., Poisson, J., and Zhang, Y. (2015). The I-TASSER Suite: protein structure and function prediction. Nat. Methods *12*, 7–8.

Young, M.M., Tang, N., Hempel, J.C., Oshiro, C.M., Taylor, E.W., Kuntz, I.D., Gibson, B.W., and Dollinger, G. (2000). High throughput protein fold identification by using experimental constraints derived from intramolecular cross-links and mass spectrometry. Proc. Natl. Acad. Sci. USA *97*, 5802–5806.

Zhang, K. (2016). Gctf: Real-time CTF determination and correction. J. Struct. Biol. *193*, 1–12.

Zheng, S.P.E., Armache, J.P., Cheng, Y., and Agard, D. (2017). MotionCor2: anisotropic correction of beam-induced motion for improved single-particle electron cryo-microscopy. Nat. Methods *14*, 331–332.

# STAR★METHODS

## KEY RESOURCES TABLE

| REAGENT or RESOURCE | SOURCE | IDENTIFIER |
|---|---|---|
| Antibodies | | |
| DYKDDDDK Tag Antibody (anti-FLAG) from rabbit | Cell Signaling | Cat#2368 |
| Anti-GFP from mouse | Roche | Cat#11814460001 |
| Monoclonal Anti-α-Tubulin antibody produced in mouse | Sigma-Aldrich | Cat#T6074 |
| Mouse TrueBlot ULTRA: Anti-Mouse Ig HRP | Rockland | Cat#18-8817-33 |
| Rabbit TrueBlot: Anti-Rabbit IgG HRP | Rockland | Cat#18-8816-33 |
| Chemicals, Peptides, and Recombinant Proteins | | |
| Lipofectamine 2000 | Thermo Fisher Scientific | Cat#11668019 |
| Protease inhibitor cocktail | Sigma-Aldrich | Cat#5056489001 |
| Benzonase Nuclease | Merck | Cat#70746-3 |
| FRET-labeled mononucleosomes | Greg Bowman Lab | N/A |
| FAM-labeled mononucleosomes | Greg Bowman Lab | N/A |
| Human PARP1 | Enzo Life Science | Cat#ALX-201-063-C020 |
| $^{32}$P-β-NAD | Perkin Elmer | Cat#NEG023X |
| Phosphate sensor | Thermo Fisher Scientific | Cat#PV4406 |
| Human PARP1 | Thermo Fisher Scientific | Cat#11040H08B50 |
| Halt protease inhibitor cocktail | Thermo Fisher Scientific | Cat#78430 |
| DSS | Creative Molecules | Cat#001S |
| Human PARP1 | Trevigen | Cat#4668-500-01 |
| Human PARP1 | BPS Bioscience | Cat#BPS-80501 |
| 99.8% $D_2O$ | ACROS | Cat#AC16630 |
| Deposited Data | | |
| Mendeley Dataset | This paper | https://doi.org/10.17632/hm7zy8kn94.1 |
| Experimental Models: Cell Lines | | |
| Human: U2OS cells | Francis Crick Cell Services | Cat#2179 |
| Experimental Models: Organisms/Strains | | |
| *E. coli* Rosetta 2 (DE3) | Novagen | Cat#71400 |
| Oligonucleotides | | |
| DNA oligonucleotides for mutagenesis | This paper | Table S2 |
| Recombinant DNA | | |
| Human ALC1$^{fl}$ (16-879) pNIC-CH2 | This paper | N/A |
| YFP-ALC1 pDEST-YFP/FRT/TO | Ahel et al., 2009 | N/A |
| Software and Algorithms | | |
| I-Tasser server | Yang et al., 2015 | https://zhanglab.ccmb.med.umich.edu/I-TASSER/ |
| GROMACS software package | Berendsen et al., 1995 | http://www.gromacs.org |
| ATSAS software package | Petoukhov et al., 2012 | https://www.embl-hamburg.de/biosaxs/software.html |
| MotionCorr | Li et al., 2013 | http://cryoem.ucsf.edu/software/driftcorr.html |
| MotionCor2 | Zheng et al., 2017 | http://msg.ucsf.edu/em/software/motioncor2.html |
| RELION | Scheres, 2012, 2015 | https://www2.mrc-lmb.cam.ac.uk/relion/index.php/Download_%26_install |
| Gctf | Zhang, 2016 | http://www.mrc-lmb.cam.ac.uk/kzhang/ |

(*Continued on next page*)

| *Continued* | | |
|---|---|---|
| REAGENT or RESOURCE | SOURCE | IDENTIFIER |
| xQuest/xProphet software pipeline | Walzthoeni et al., 2012 | http://proteomics.ethz.ch/cgi-bin/xquest2_cgi/index.cgi |
| DynamX HDX Data Analysis Software | Waters | Cat#720005145en |
| Other | | |
| 5 mL HisTrap FF | GE Healthcare | Cat#17525501 |
| HiPrep 26/10 Desalting | GE Healthcare | Cat#17508701 |
| 5 mL HiTrap Q HP | GE Healthcare | Cat#17115401 |
| 5 mL HiTrap SP HP | GE Healthcare | Cat#17115201 |
| HiLoad 16/600 Superdex 200 pg | GE Healthcare | Cat#28989335 |
| Superdex 200 10/300 GL (discontinued) | GE Healthcare | Cat#17517501 |
| Gel Filtration Markers Kit | Sigma-Aldrich | Cat#MWGF1000 |
| ANTI-FLAG M2 Affinity Gel | Sigma-Aldrich | Cat#A2220 |
| GFP-Trap_A | Chromotek | Cat#gta-20 |
| Bio SEC-3 | Agilent | Cat#5190-6854 |
| Ni-NTA Agarose resin | QIAGEN | Cat#30210 |
| Enzymate BEH pepsin column | Waters | Cat#186007233 |
| ACQUITY UPLC BEH C18 VanGuard Pre-column | Waters | Cat#186003975 |
| ACQUITY UPLC BEH C18 Column | Waters | Cat#186002350 |

## CONTACT FOR REAGENT AND RESOURCE SHARING

Further information and requests for resources and reagents should be directed to and will be fulfilled by the lead contact, Sebastian Deindl (sebastian.deindl@icm.uu.se).

## EXPERIMENTAL MODEL AND SUBJECT DETAILS

cDNAs used for expression of ALC1 recombinant protein were of *Homo sapiens* origin.

Cell lines as well as growth and culture conditions are described in the method details.

## METHOD DETAILS

### Cloning, Plasmids, and Cell Lines

ALC1$^{cat}$ (residues 16-612), ALC1$^{macro}$ (residues 613-879), ALC1$^{\Delta N}$ (residues 253-879), and mutant ALC1$^{fl}$ constructs were generated by PCR-based or site-directed mutagenesis of the ALC1$^{fl}$ construct encoding a 6xHis-tagged version of human ALC1 (residues 16–879). For the ALC1$^{macro\Delta 6xHis}$, a TEV-cleavage site was added to the ALC1$^{macro}$ construct. For *in vivo* experiments, an YFP-labeled construct of ALC1 (YFP-ALC1 pDEST-YFP/FRT/TO) previously generated by amplification from a human HeLa cDNA library and subsequent cloning by Gateway LR reaction (Ahel et al., 2009) was used. Mutations in the N- and C-terminal ATPase lobe as well as the macro domain were introduced using site-directed mutagenesis. U2OS cells we transiently transfected using Lipofectamine 2000 (Thermo Fisher) following the manufacturer's instructions.

### Protein Production and Purification

Proteins were expressed in Rosetta 2 (DE3) cells (Novagen). Protein expression was induced with 0.5 mM IPTG at $OD_{600} = 2.0$ at 18°C overnight. Cell pellets were resuspended in a buffer containing 20 mM HEPES, pH 7.5, 500 mM NaCl, 20 mM imidazole, 10% glycerol, 5 mM β-mercaptoethanol, a protease inhibitor cocktail (Sigma-Aldrich) and Benzonase Nuclease (Merck Millipore). The cells were lysed using a sonicator, centrifuged, and the resulting supernatant was filtered through a 0.45 μm filter and loaded onto a 5 mL HisTrap FF column (GE Healthcare). The protein was eluted using a linear imidazole gradient (20–500 mM). Fractions were pooled and desalted using a HiPrep 26/10 desalting column (GE Healthcare) and the following buffer: 20 mM MES pH 6.0, 300 mM NaCl, 10% glycerol, 1 mM DTT (for ALC1$^{fl}$); 20 mM MES pH 6.9, 200 mM NaCl, 10% glycerol, and 1 mM DTT (for ALC1$^{\Delta N}$); 20 mM Tris pH 7.5, 200 mM NaCl, 10% glycerol, 1 mM DTT (for ALC1$^{cat}$); 20 mM HEPES pH 8, 200 mM NaCl, 10% glycerol, 1 mM DTT (ALC1$^{macro}$). The protein was loaded onto a subtractive 5 mL Q HP column (GE Healthcare) attached in tandem with a 5 mL SP HP column (GE Healthcare) (for ALC1$^{fl}$, ALC1$^{\Delta N}$, and ALC1$^{macro}$) or onto a 5 mL Q HP column (for ALC1$^{cat}$) and eluted with a linear salt gradient (300-1000 mM NaCl for ALC1$^{fl}$ and 200-1000 mM for all other constructs). Fractions were concentrated and injected

onto a HiLoad 16/600 Superdex 200 pg column (GE Healthcare) equilibrated with 20 mM MES pH 6.5, 300 mM NaCl, 10% glycerol, and 0.5 mM TCEP. The final protein was concentrated to typically 10 mg/mL, flash frozen in liquid nitrogen, and stored at −80°C.

For the ALC1$^{macro\Delta 6xHis}$ construct, TEV cleavage of the 6xHis tag was performed over night at 4°C. The protein was then loaded onto the HisTrap FF column again and the flow through and wash were collected and desalted as above.

## Dynamic Light Scattering (DLS)

Experiments were carried out using a W130i DLS system (Avid Nano) at 4°C and a protein concentration of approximately 7 mg/mL. The buffer used was 20 mM MES pH 6.5, 300 mM NaCl, 10% glycerol, and 0.5 mM TCEP.

## PAR Binding Assay

For *in vitro* ADP-ribosylation, 200 ng of recombinant PARP1 (Enzo Life Science) were suspended in 200 μL reaction buffer (100 mM Tris pH 8, 10 mM $MgCl_2$, 1 mM DTT). Fourty nM β-NAD and 60 nM $^{32}$P-β-NAD (Perkin Elmer) were added, the reaction was incubated for 30 min at 37°C while shaking, and stopped by Proteinase K digestion at 42°C for 1 h. Resulting poly(ADP-ribose) polymers were subjected to phenol-chloroform-extraction, resuspended in TBS-T (Tris buffered saline - 0.1% TWEEN), and stored at −20°C.

For the dot-blot, a native nitrocellulose membrane was soaked in TBS-T for 10 min and left to air-dry. Two, 4, 8, and 16 pmol of ALC1 and BSA were dot-blotted onto the nitrocellulose membrane and the blot was left to dry for 2 h. Upon blocking for 1 h in TBS-T containing 10% fatty-acid free milk, the membrane was incubated in poly(ADP-ribose) polymer solution at 4°C overnight. The next day the membrane was washed with TBS-T containing 150 μM NaCl for 1 h and exposed to Fuji-X X-ray film for 5 days before imaging with a Fuji-X Bio-Imager.

## ATPase Activity Measurements

ATPase activity was measured as described previously (Ahel et al., 2009) by monitoring the fluorescence intensity of a phosphate binding protein (Brune et al., 1994; Ferreira et al., 2007) labeled with a coumarin-based fluorescent dye, 7-diethylamino-3-((((2-maleimidyl)ethyl)amino)carbonyl) coumarin (MDCC), as a readout of the amount of inorganic phosphate ($P_i$) generated by ATP hydrolysis. Upon binding $P_i$, the fluorescence of the labeled phosphate binding protein (MDCC-PBP) increases and its emission wavelength shifts. The increase in fluorescence was recorded in solution using a Spark 10 M (Tecan) instrument. 25 μL ATPase reactions were carried out in 50 mM Tris pH 7.9, 50 mM NaCl, and 5 mM $MgCl_2$ buffer with 80 nM ALC1 and 1 mM ATP. Where indicated, 50 μM $NAD^+$ and 80 nM PARP1 (Thermo Fisher) and/or 120 nM 239 bp dsDNA were added. Upon incubation for 2 h at 37°C, phosphate sensor (Thermo Fisher) was added at a final concentration of 0.5 μM and immediately measured (excitation = 420 nm, emission = 465 nm).

## Restriction Enzyme Accessibility Assay for Nucleosome Remodeling

In a reaction volume of 10 μl, 1 or 10 pmol ALC1$^{fl}$ or ALC1$^{cat}$ were incubated with 0.2 pmol Cy3/Cy5-labeled mononucleosomes for 30 min at 32°C in buffer containing 20 mM HEPES, pH 7.9, 50 mM NaCl, 4.5 mM $MgCl_2$, 10% glycerol, 0.02% Triton X-100, 2 mM DTT, 45 μg/mL BSA, and 2 mM ATP. Where indicated, reactions contained 1 pmol PARP1 (Thermo Fisher) pre-incubated with 50 μM $NAD^+$ for 5 min at 37°C. Reaction products were incubated with 10 U HhaI for 30 min at 37°C. Histone proteins were degraded by incubation with 1 mg/mL Proteinase K for 1 h at 37°C and the samples were analyzed on a 15% polyacrylamide/8 M urea gel that was imaged for Cy5.

## FRET Assay for Nucleosome Remodeling

Remodeling kinetics of nucleosomes were measured by monitoring the total acceptor (Cy5) signal (under a constant 532 nm illumination that excited the donor Cy3) of FRET-labeled mononucleosomes after addition of ALC1/ATP and, when indicated, PARP1/$NAD^+$ to initiate remodeling. As nucleosomes were moved away from the end of the DNA, the FRET efficiency decreased, which resulted in a decrease in the Cy5 signal.

The nucleosome remodeling assay was performed by diluting nucleosomes in remodeling buffer (40 mM Tris pH 7.5, 12 mM HEPES pH 7.9, 60 mM KCl, 0.32 mM EDTA, 3 mM $MgCl_2$, 100 μg $mL^{-1}$ acetylated BSA, 10% glycerol, 0.02% IGEPAL CA-630, 10% glucose, 2 mM Trolox) in a cuvette fitted for a Horiba Jovin Yvon Fluorolog spectrometer. Immediately before starting a kinetic scan (excitation = 532 ± 10 nm, emission = 670 ± 14.7 nm, 1 Hz acquisition frequency), 80 nM ALC1 (for ALC1$^{complex}$, 80 nM ALC1$^{cat}$ and 80 nM ALC1$^{macro}$ were mixed), 1 mM ATP and additional 1 mM $MgCl_2$ were mixed in the remodeling buffer and added to the nucleosomes, achieving a final concentration of 3.7 nM nucleosomes in a final reaction volume of 100 μl. Where indicated, 80 nM PARP1 (Thermo Fisher) preincubated with 50 μM $NAD^+$ and 120 nM 239 bp dsDNA for 5 min at 37°C, was added to the reaction. Remodeling time courses were filtered (5-point adjacent-averaging) and fit to an exponential decay curve, $y = A1*\exp(-kx)+y_0$, where k is the remodeling rate for a given time course. The bar plots show relative remodeling rates as indicated.

## Reconstitution of ALC1$^{complex}$

7 nmol of either ALC1$^{fl}$, ALC1$^{cat}$, ALC1$^{macro}$, or in case of ALC1$^{complex}$ 7 nmol of each ALC1$^{cat}$ and ALC1$^{macro}$, were incubated on ice for 10 min, spun down for 10 min at 4°C and 13,000 × g, and injected onto a Superdex 200 10/300 GL column. Each run was performed at a flowrate of 0.2 mL/min with a buffer containing 20 mM MES, pH 6.5, 10% glycerol, 0.5 mM TCEP and either 500 mM NaCl

(for ALC1$^{cat}$ and ALC1$^{macro}$) or 300 mM NaCl (for ALC1$^{fl}$ and ALC1$^{complex}$). Peak fractions were collected and 3 $\mu$g of each protein sample was analyzed by SDS-PAGE.

The calibration of the Superdex 200 10/300 GL column was carried out with Blue Dextran (void volume) $\beta$-Amylase from sweet potato (200 kDa), Alcohol dehydrogenase from yeast (150 kDa), Bovine serum albumin (66 kDa), and Carbonic anhydrase from bovine (29 kDa) from the Gel Filtration Markers Kit (Sigma-Aldrich) according to manufacturer's instructions.

## Electrophoretic Mobility Shift Assay (EMSA)

0.01, 0.1, 1, 1.3, 1.7, 2, 4, 8, and 16 $\mu$M ALC1$^{macro}$ were incubated with 200 nM of either 239 bp dsDNA, 0-601-11, or 40-601-40 FAM-labeled mononucleosomes for 90 min at room temperature. The reaction volume was 10 $\mu$l in a buffer containing 10 mM HEPES pH 7.9, 50 mM NaCl, 3 mM $MgCl_2$, 5% glycerol, 1 mM DTT, and 0.25 mg/mL BSA. The entire reaction was loaded onto a 4%–20% gradient polyacrylamide gel and run for 90 min at 100 V in 0.25x TBE buffer. The DNA gel was stained with EtBr and imaged under UV, while the two nucleosome gels were imaged using FAM fluorescence emission.

## Homology Modeling

Automated homology modeling of the N-terminal ATPase, C-terminal ATPase, and macro domains of ALC1 was performed by the I-Tasser server (Yang et al., 2015). Only regions of ALC1 with homologous proteins in the Protein Data Bank were modeled: residues 32-264 for the ALC1 N-terminal ATPase lobe, residues 271-545 for the C-terminal ATPase lobe, and residues 716-879 for the macro domain. The top-scoring models from the three predictions had final C-scores of 1.12, 1.25 and 0.49 for the N-terminal ATPase, C-terminal ATPase, and macro domain, respectively. The C-score is used as a confidence score for predicted models by the I-Tasser server and increases from $-5$ to 2 with increasing confidence in the model.

## Molecular Dynamics Simulations

The three homology models (N-terminal ATPase, C-terminal ATPase, and macro domain) were individually prepared for molecular dynamics (MD) simulations by solvation in orthorhombic boxes with periodic boundary conditions. Ion concentrations of 50 mM NaCl and 5 mM $MgCl_2$ were used, where additional $Na^+$ or $Cl^-$ ions were added as counter ions for neutralization. The size of the simulation box was chosen so that at least 1 nm of water was present between the protein and the edge of the box. After preparation of the system all MD simulations were performed with the GROMACS software package (Berendsen et al., 1995; Van der Spoel et al., 2005) version 5.1. All three systems were energy minimized using a steepest descent algorithm until the maximum force on any atom in the system was smaller than 100 kJ $mol^{-1}$ $nm^{-1}$. The systems were subsequently relaxed and temperature equilibrated by performing 200 ps simulations at constant volume, followed by 200 ps simulations at constant pressure. Production MD simulations were carried out at constant pressure in duplicates for each structure, generating a total of 6 trajectories lasting 100 ns. The initial velocities in the production simulations were generated according to the Maxwell-Boltzmann distribution using a different random number seed for each trajectory.

## Small Angle X-Ray Scattering (SAXS)

Data collection and data reduction: Batch-mode SAXS data for ALC1$^{fl}$ were collected at beamline BM29 (European Synchrotron Radiation facility, Grenoble, France). Prior to data collection, the protein sample was centrifuged at 13,000 rpm and 4°C for 10 min and diluted in a 2-fold dilution series starting at approximately 10 mg/mL. The final protein concentrations were determined as 10.8, 5.3, 2.6 and 1.2 mg/mL. For each protein concentration, 10 frames with 1 s exposure time were collected at 4°C. Data were collected on a Pilatus 1M detector at 2.867 m distance, enabling measurements with an angular q range of 0.025 - 5 $nm^{-1}$. Data from buffer samples (20 mM MES pH 6.5, 300 mM NaCl, 10% glycerol, 0.5 mM TCEP) used for background subtraction were collected both before and after measuring the protein samples. Primary data reduction (scaling, averaging, and background subtraction), calculation of radius of gyration ($R_g$) and maximum particle distance ($D_{max}$) were performed by the ATSAS package (Petoukhov et al., 2012).

For in-line SEC measurements of ALC1$^{\Delta N}$, ALC1$^{cat}$, and ALC1$^{fl}$, or for batch measurements of ALC1$^{cat}$ in the presence of 1.1x molar excess of 25 bp dsDNA, SAXS data were collected at beamline B21 (Diamond Light Source, Didcot, UK) using an Agilent 1200 HPLC system equipped with a 5 mL BioSEC-3 column (Agilent) for ALC1$^{\Delta N}$ and a Superdex 200 10/300 column (GE Healthcare) for ALC1$^{cat}$ and ALC1$^{fl}$. Prior to data collection, the columns were equilibrated with 20 mM MES pH 6.5, 300 mM NaCl, 10% glycerol, 0.5 mM TCEP. The protein stock was centrifuged at 13,000 rpm for 10 min at 4°C. 45 $\mu$L of the sample at a concentration of 13.8 mg/mL for ALC1$^{\Delta N}$, 8.4 mg/mL for ALC1$^{cat}$, and 8.8 mg/mL for ALC1$^{fl}$, were loaded onto the size exclusion column and data were collected continuously during the sample elution. Data were recorded at an energy of 12.4 keV on a Pilatus 2M detector and at a distance of 4.014 m, allowing the collection of an angular q range between 0.038–4.2 $nm^{-1}$. Primary data reduction (scaling, averaging, and background subtraction) was performed with ScÅtter (http://www.bioisis.net/tutorial/) using data frames corresponding to the protein monomeric peak. Only frames which showed stable frame-by-frame $R_g$ determinations were used in data reduction. The radius of gyration ($R_g$) and maximum particle distance ($D_{max}$) were calculated using the ATSAS package (Petoukhov et al., 2012).

Shape reconstructions and SAXS-based rigid-body modeling: For ALC1$^{fl}$, all modeling was based on the data measured in batch mode at a concentration of 1.2 mg/mL. For ALC1$^{\Delta N}$, the in-line SEC data were used for modeling. *Ab initio* shape reconstructions were generated using the program GASBOR (Svergun et al., 2001) and the X-ray scattering profile, $D_{max}$, as well as the number of residues (dummy atoms) to be modeled as input. GASBOR calculations were carried out 20 times using scattering data to $q_{max}$

of 0.399 Å$^{-1}$. Individual shape reconstructions exhibited overall similar shapes, and representative molecular envelopes are shown. Two/three-domain rigid-body models of ALC1$^{\Delta N}$, ALC1$^{cat}$ and ALC1$^{fl}$, were reconstructed from the SAXS data using the program BUNCH (Petoukhov and Svergun, 2005). In our case, the domain homology models were used to compute a theoretical scattering pattern. The unknown regions not included in the domain homology models were represented by an appropriate number (one per amino acid residue) of dummy residues. BUNCH calculates the relative domain positions and orientations as well as conformations of the dummy residue chains that are most consistent with the SAXS data. Fits obtained for the computed scattering profiles to the experimental SAXS data exhibited major deviations only in the high-resolution range, for q > 0.3 Å$^{-1}$. We performed 20 replicates of the BUNCH rigid-body modeling procedure.

Rigid-body models for ALC1$^{fl}$: All models exhibited overall similar shapes, but could be divided into two main groups based on whether the macro domain was placed against the C-terminal ATPase domain (ALC1$^{fl}$-MC) or the N-terminal ATPase domain (ALC1$^{fl}$-MN), with approximately 75% and 25% in the ALC1$^{fl}$-MC and ALC1$^{fl}$-MN groups, respectively. Within each group, models exhibited highly similar structures with near-identical translational positions and only minor differences in the rotational orientation of the three domains (Figure S2C). The ALC1$^{fl}$-MC and ALC1$^{fl}$-MN structures shown in Figure 2C are representative of the dominant cluster of similar structures.

Rigid-body models for ALC1$^{cat}$ and ALC1$^{\Delta N}$: All models were essentially identical in the translational position of domains but deviated slightly in their relative rotational orientation. A structure representative of the largest cluster of similar structures is shown.

Interdomain cross-links: Where indicated in the text, subsets of three Macro:C-ATPase and/or Macro:N-ATPase cross-links each were used as restraints during rigid-body modeling trials. Restraints were defined as contact conditions in BUNCH using the default contact penalty weight and a distance threshold of 40 Å.

## Sample Preparation for Electron Microscopy, Data Collection, and Analysis

Negative-stain electron microscopy: 3 μL aliquots of purified ALC1$^{fl}$ at a concentration of 150 nM were placed on freshly glow-discharged carbon-coated grids and incubated for 1 min. Excess liquid was blotted away and 20 μL of 2% (w/v) uranyl acetate solution was applied immediately. A small amount of the stain solution was allowed to incubate on the grid for 1 min, then blotted away and dried. 1.5 s exposure images at a nominal magnification of × 92,000 (yielding a pixel size of 1.54 Å) were recorded on a Talos Arctica (FEI) electron microscope operating at 200 kV and equipped with a Falcon-II detector using automated data acquisition software (EPU).

Images were processed using MOTIONCORR (Li et al., 2013) for whole-frame motion correction and phase-flips were applied at the stage of CTF estimation by Gctf (Zhang, 2016). Particles (~44,000) were automatically picked using Gautomatch and subjected to reference-free 2D classification in RELION (Scheres, 2012, 2015).

Electron cryo-microscopy: 3 μL aliquots of purified ALC1$^{fl}$ at a concentration of 150 nM were placed on holey carbon grids (Quantifoil Au R1.2/1.3, 300 mesh) pre-coated with graphene oxide, and flash frozen in liquid ethane using a manual plunger. Zero-energy-loss images were recorded manually on an FEI Titan Krios electron microscope at 300 kV, using a slit width of 20 eV on a GIF-Quantum energy filter. A Gatan K2-Quantum detector was used in super-resolution counting mode at a nominal magnification of × 165,000 (yielding a pixel size of 0.83 Å). The total dose on the specimen was ~43 e$^{-}$/Å$^{2}$ fractionated into 20 movie frames over 5 s (a dose rate of 4.8 e$^{-}$/pixel/s) and the defocus values in the final dataset ranged from 0.6 to 3.5 μm.

Cryo-EM images were processed using Motioncor2 (Zheng et al., 2017) for patched frame motion correction and dose-weighting, Gctf (Zhang, 2016) was used for estimation of the contrast transfer function parameters, and RELION for all other image processing steps. Templates for reference-based particle picking were obtained from 2D class averages that were calculated from a manually picked subset of the micrographs. Using low-pass filtered templates to 20 Å resolution to limit reference bias, ~47,000 particles were picked automatically from a total of 250 micrographs that were manually cleaned of false-positives yielding a dataset of ~40,000 particles. Two rounds of reference-free 2D classification were employed to generate the presented classes.

## Cross-Linking Coupled to Mass Spectrometry (XL-MS)

Chemical cross-linking of purified ALC1$^{fl}$ was performed in 20 mM HEPES pH 7.4, 300 mM NaCl, 10% glycerol, 0.5 mM TCEP using a 1:1 mixture of deuterated ($d_{12}$) and nondeuterated ($d_0$) disuccinimidyl suberate (DSS, Creative Molecules Inc) as a cross-linking agent. Preliminary cross-linking experiments indicated oligomer formation at higher protein concentrations (Figure S2E). Final cross-linking reactions therefore contained 0.5 mM DSS and 0.15 mg/mL ALC1$^{fl}$. Under these conditions, only monomeric ALC1$^{fl}$ could be detected by SDS-PAGE. The cross-linking reaction was carried out for 30 min at 37°C and stopped by adding 1 M aqueous ammonium bicarbonate solution to a final concentration of 50 mM. After further incubation for 30 min at 37°C, all samples were evaporated to dryness in a vacuum centrifuge. Cross-linked samples were reduced, alkylated, and digested in solution using endoprotease Lys-C and trypsin. Cross-linked peptides were purified by solid-phase extraction on Sep-Pak tC18 cartridges (Waters), fractionated by size-exclusion chromatography, and MS analyses and cross-link assignments were carried out essentially as described previously (Leitner et al., 2012) using an Orbitrap Elite mass spectrometer (Thermo Fisher) and the xQuest/xProphet software pipeline (Walzthoeni et al., 2012).

Detected cross-links were mapped onto the given structural model (ALC1$^{fl}$-MC or ALC1$^{fl}$-MN) and, based on distances between corresponding residues, could be divided into two clusters. The first cluster featured large distances of ~90.0 Å (median value), substantially larger than the generally accepted maximum predicted distance of 30 Å between two lysine residues cross-linked by DSS,

prompting us to identify these cross-links as "non-plausible." The second cluster exhibited distances of ~45.4 Å (median value), much shorter than the median distance of 90.0 Å for the non-plausible cross-links. Cross-links of the second cluster were therefore identified as "plausible." We note that the "plausible" cluster involved, next to distances below or around 30 Å, a number of distances larger than 30 Å. These distances were still substantially smaller than all distances of the non-plausible cluster and likely due to local flexibility. Small-scale transient changes can yield short-lived deviations from the "average" structure with slightly shortened distances that could permit cross-linking. Indeed, the conformation of Snf2-type motor proteins is anticipated to be somewhat dynamic (Narlikar et al., 2013).

Sample preparation for $ALC1^{fl}$ in the presence of non-PARylated PARP1 (non-activated) or PARylated PARP1 (activated): In a final reaction volume of 100 μl, 1.75 μM PARP1 (Trevigen) were incubated with 2.6 μM 239 bp dsDNA and 1.1 mM $NAD^+$ for 30 min at 25°C in a buffer containing 50 mM HEPES pH 7.9, 50 mM NaCl, 5 mM $MgCl_2$. After another 10 min at room temperature, 1.75 μM $ALC1^{fl}$ and 1 mM ATP were added to the reaction. For the non-activated sample, $NAD^+$ was omitted. Both samples were incubated at room temperature for 10 min prior to the cross-linking reaction.

### Purification of In *Trans* Complex over Ni-NTA Resin

5 μM of either $ALC1^{cat}$, or $ALC1^{macro6x\Delta His}$, or in case of $ALC1^{complex}$ 5 μM of both proteins were incubated on ice for 10 min in a volume of 50 μl. Where indicated, 5 μM GST-tagged PARP1 (BPS Bioscience) was incubated with 7.5 μM 230 bp dsDNA and 3125 μM $NAD^+$ for 5 min at 37°C before adding to the complex. The 50 μl reactions were mixed with Ni-NTA agarose resin (QIAGEN) pre-equilibrated in washing buffer (20 mM HEPES pH 7.5, 300 mM NaCl, 10% glycerol, 20 mM imidazole, 0.5 mM TCEP). After 1 h at 4°C the resin was pelleted and the supernatant was kept as "flow-through" (F). After the resin was washed twice with 800 μl washing buffer for 5 min at 4°C, 50 μl of elution buffer (20 mM HEPES pH 7.5, 500 mM NaCl, 10% glycerol, 500 mM imidazole, 0.5 mM TCEP) were added to the resin an incubated at room temperature for 30 min. The resin was pelleted and the supernatant was kept as "elution" (E). 10 μl of F and E for each reaction were analyzed by SDS-PAGE.

### Immunoprecipitation (IP)

U2OS cells were seeded at $2 \times 10^6$ in a 10-cm dish and transfected with 2.5 μg of "bait" and 2.5 μg of "prey" DNA 24 h later. Following another 24 h, cells were scraped in ice cold PBS and lysed in IP buffer (50 mM Tris, 50 mM NaCl, 2 mM $CaCl_2$, 2 mM $MgCl_2$, 0.1% TWEEN 20 and 0.5% Triton X-100) supplemented with 1x Halt Protease & Phosphatase inhibitor cocktail (Thermo Fisher) and sonicated using a microprobe for 5 s on ice. Samples were then centrifuged for 10 min at 17,900 × g at 4°C and transferred to a new tube.

Lysates were incubated with prewashed and equilibrated anti-FLAG M2 affinity gel (FLAG-IP) or GFP-Trap_A agarose beads (YFP-IP) overnight at 4°C with constant rotation. Beads were washed twice with IP buffer then mixed with sample buffer and boiled at 100°C for 5 min in the presence of 2.5% β-ME before being subjected to SDS-PAGE and immunoblot. To demonstrate the levels of proteins in cell lysates used for immunoprecipitation, 15% of cell lysates were loaded per lane and indicated as "Input." DYKDDDDK tag antibodies from rabbit (Cell Signaling), α-GFP antibodies from mouse (Roche), and monoclonal α-Tubulin antibody (Sigma-Aldrich) from mouse were used as primary antibodies. Mouse and Rabbit Trueblot secondary antibodies (Rockland) were used to minimize unwanted background associated with IP.

### Hydrogen-Deuterium Exchange Mass Spectrometry (HDX-MS)

Deuterium exchange reactions of $ALC1^{fl}$ with and without activated (PARylated) and non-activated PARP1 were initiated by diluting the protein in $D_2O$ (99.8% $D_2O$ ACROS) in 50 mM Tris, 50 mM NaCl, 5 mM $MgCl_2$, 1.5 μM 239 bp dsDNA, 1mM ATP, 1mM TCEP pH 7.9 buffer to give a final $D_2O$ percentage of ~70%. For all experiments, deuterium labeling was carried out at 23°C (unless otherwise stated) at five time points (3 s on ice (0.3 s), 3 s, 30 s, 300sec, and 3000sec) in triplicates. The labeling reaction was quenched by the addition of chilled 2.4% (v/v) formic acid in 2 M guanidinium hydrochloride and snap frozen in liquid nitrogen. Samples were stored at −80°C prior to analysis.

The quenched protein samples were rapidly thawed and subjected to enzymatic digestion by pepsin followed by reversed phase UPLC separation (Mevissen et al., 2016). Briefly, the protein was passed through an Enzymate BEH immobilized pepsin column, 2.1 × 30 mm, 5 μm (Waters, UK) at 200 μL/min for 2 min and the peptic peptides trapped and desalted on a 2.1 × 5 mm C18 trap column (Acquity BEH C18 Van-guard pre-column, 1.7 μm, Waters, UK). Trapped peptides were subsequently eluted with an 11 min gradient of acetonitrile in 0.1% v/v formic acid (3%–43%) at 40 μL/min. Peptides were separated on a reverse phase column (Acquity UPLC BEH C18 column 1.7 μm, 100 mm x 1 mm, Waters, UK), and detected on a SYNAPT G2-Si HDMS mass spectrometer (Waters, UK) acquiring over a *m/z* of 300 to 2000, with the standard electrospray ionization (ESI) source and lock mass calibration using [Glu1]-fibrino peptide B (50 fmol/μL). The mass spectrometer was operated at a source temperature of 80°C and a spray voltage of 2.6 kV. Peptide identification was performed by $MS^e$ (Silva et al., 2005) and the resulting $MS^e$ data analyzed using Protein Lynx Global Server software (Waters, UK) with an MS tolerance of 5 ppm. Mass analysis of the peptide centroids was carried out with the DynamX software (Waters, UK). Only peptides with a score > 6.4 were considered. In the first instance, analysis and identification was performed automatically, however, all peptides (deuterated and non-deuterated) were manually verified at every time point for the correct charge state, presence of overlapping peptides, and correct retention time. Deuterium incorporation was not corrected for back-exchange.

### Laser Micro-irradiation Induced DNA Damage

In order to detect local recruitment of YFP-tagged ALC1 to laser-induced DNA damage U2OS were seeded on 35 mm glass bottom dishes. Cells were pre-sensitized with 10 μM BrdU and transfected with YFP-ALC1 24 h prior to imaging. Cells were transferred to an Olympus FV1000 confocal laser-scanning microscope with heated stage. Laser micro-irradiation was performed with a 405 nm laser focused through a 40x objective (400 mW at objective, 50 scans). To ensure that cells with similar expression levels are assayed and that YFP stayed within the dynamic detection range, cells exhibiting moderate expression levels were systematically chosen using identical 488 nm laser settings. The association and dissociation kinetics of YFP-tagged ALC1 at sites of laser micro-irradiation were monitored by measuring YFP fluorescence over time in the damaged region of interest using the 488 nm laser. Variations in fluorescence intensity (I) were plotted as a function of time (t) using GraphPad Prism7 software. For mathematical modeling of protein mobility, the association kinetics were determined from $(I_t\text{-}I_0)/(I_{max}\text{-}I_0)$ plotted as a function of time, and the dissociation kinetics from $(I_t\text{-}I_{min})/(I_{max}\text{-}I_{min})$ plotted as a function of time, where $I_{max}$ and $I_{min}$ are the maximum and mimimum values measured, respectively. Curve fitting and determination of the time constants (τ) was carried out with Prism7 software, based on a single-phase exponential model.

## QUANTIFICATION AND STATISTICAL ANALYSIS

For ATPase activity measurements, values reported represent the mean ± SEM from 3-5 independent experiments with 3 technical replicates each. For nucleosome remodeling time courses, each experimental condition was recorded in at least 3 independent experiments, and representative remodeling time courses are shown. IP data are representative of two to three independent experiments. For association/dissociation kinetics from live-cell imaging, error bars represent SEM for n = 15 cells.

## DATA AND SOFTWARE AVAILABILITY

Unprocessed images were uploaded as a Mendeley Dataset: https://doi.org/10.17632/hm7zy8kn94.1.

**Molecular Cell, Volume *68***

## Supplemental Information

# Mechanistic Insights into Autoinhibition of the Oncogenic Chromatin Remodeler ALC1

**Laura C. Lehmann, Graeme Hewitt, Shintaro Aibara, Alexander Leitner, Emil Marklund, Sarah L. Maslen, Varun Maturi, Yang Chen, David van der Spoel, J. Mark Skehel, Aristidis Moustakas, Simon J. Boulton, and Sebastian Deindl**

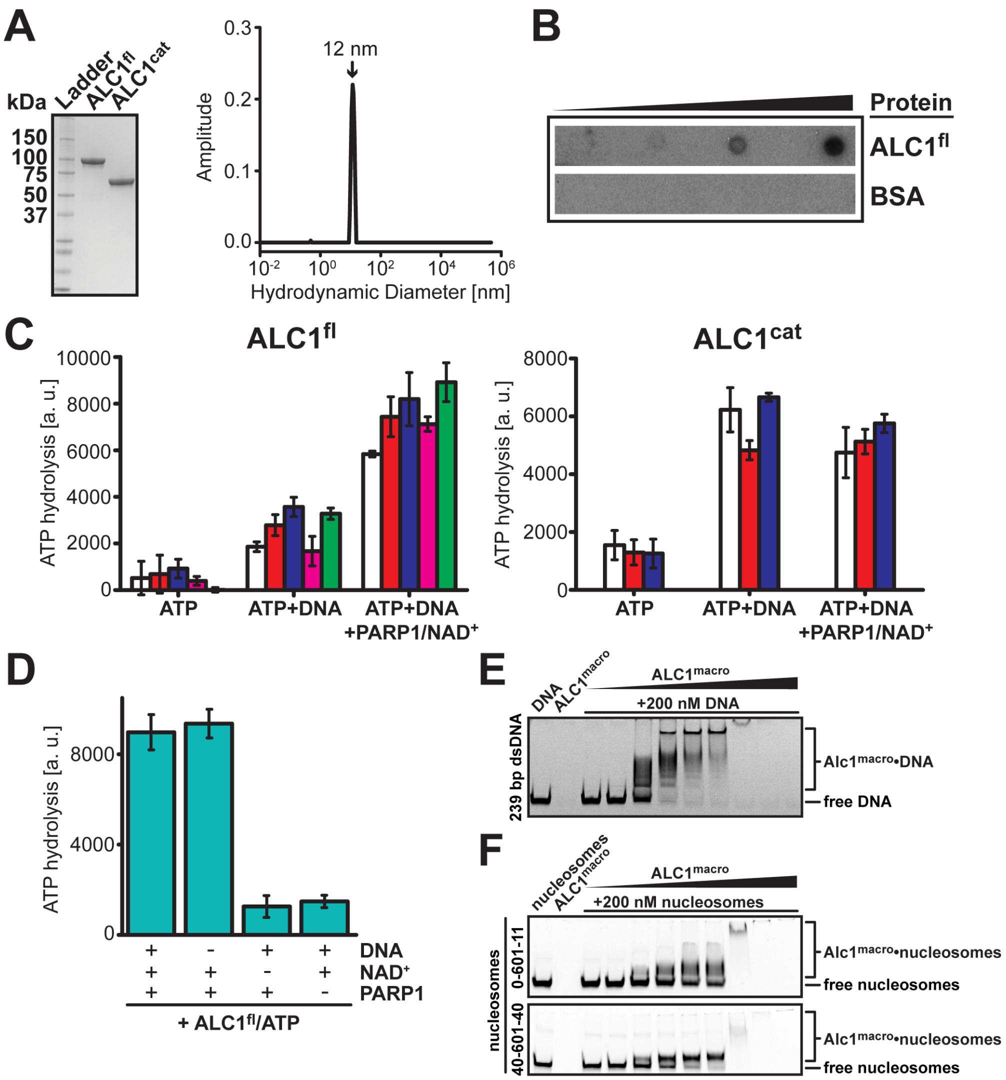


**Figure S1. Related to Figure 1. Biochemical and biophysical characterization of ALC1.**

(**A**) Left: SDS–PAGE analysis of ALC1$^{fl}$ (~98.8 kDa; lacks only 14 residues at the N terminus and 18 residues at the C terminus) and ALC1$^{cat}$ (~68.6 kDa; lacks the C-terminal macro domain and a portion of the linker, leaving only the ATPase motor and a 67-residue portion of the N terminus of the linker). Right: Dynamic light scattering (DLS) measurements with ALC1$^{fl}$. The size distribution by amplitude features a single peak at a hydrodynamic diameter of ~12 nm.

(**B**) Poly(ADP-ribose) binding assay for ALC1$^{fl}$. 2, 4, 8, and 16 pmol of ALC1$^{fl}$ or BSA were dot-blotted onto a nitrocellulose membrane and incubated with $^{32}$P-labeled PAR.

(**C**) Independent replicate experiments for ALC1$^{fl}$ (left) and ALC1$^{cat}$ (right) used to generate Figure 1A. Different colors represent the different repeats. Where indicated, PARP1/NAD$^{+}$ and/or 239 bp double-stranded (ds) DNA were added. Error bars represent SEM ($N$ = 3 technical replicates).

(**D**) Negative controls for the PARP1/NAD$^{+}$ reaction in Figure 1A. All controls were conducted with ALC1$^{fl}$/ATP either in the presence or absence of PARP1, NAD$^{+}$, or 239 bp dsDNA. Error bars represent SEM ($N$ = 3 independent experiments with 3 technical replicates each).

(**E**) Electrophoretic mobility of dsDNA (239 bp, 200 nM) in the absence or presence of 0.01, 0.1, 1, 1.3, 1.7, 2, 4, 8, and 16 µM ALC1 macro domain (ALC1$^{macro}$). As a comparison, lane 2 shows 16 µM ALC1$^{macro}$ without dsDNA.

(**F**) Electrophoretic mobility of 200 nM FAM-labeled end-positioned (0-601-11; top gel) or center-positioned (40-601-40; bottom gel) nucleosomes in the absence or presence of 0.01, 0.1, 1, 1.3, 1.7, 2, 4, 8, and 16 µM ALC1$^{macro}$. As a comparison, lane 2 shows 16 µM ALC1$^{macro}$ without nucleosomes. Both gels were imaged using FAM fluorescence.

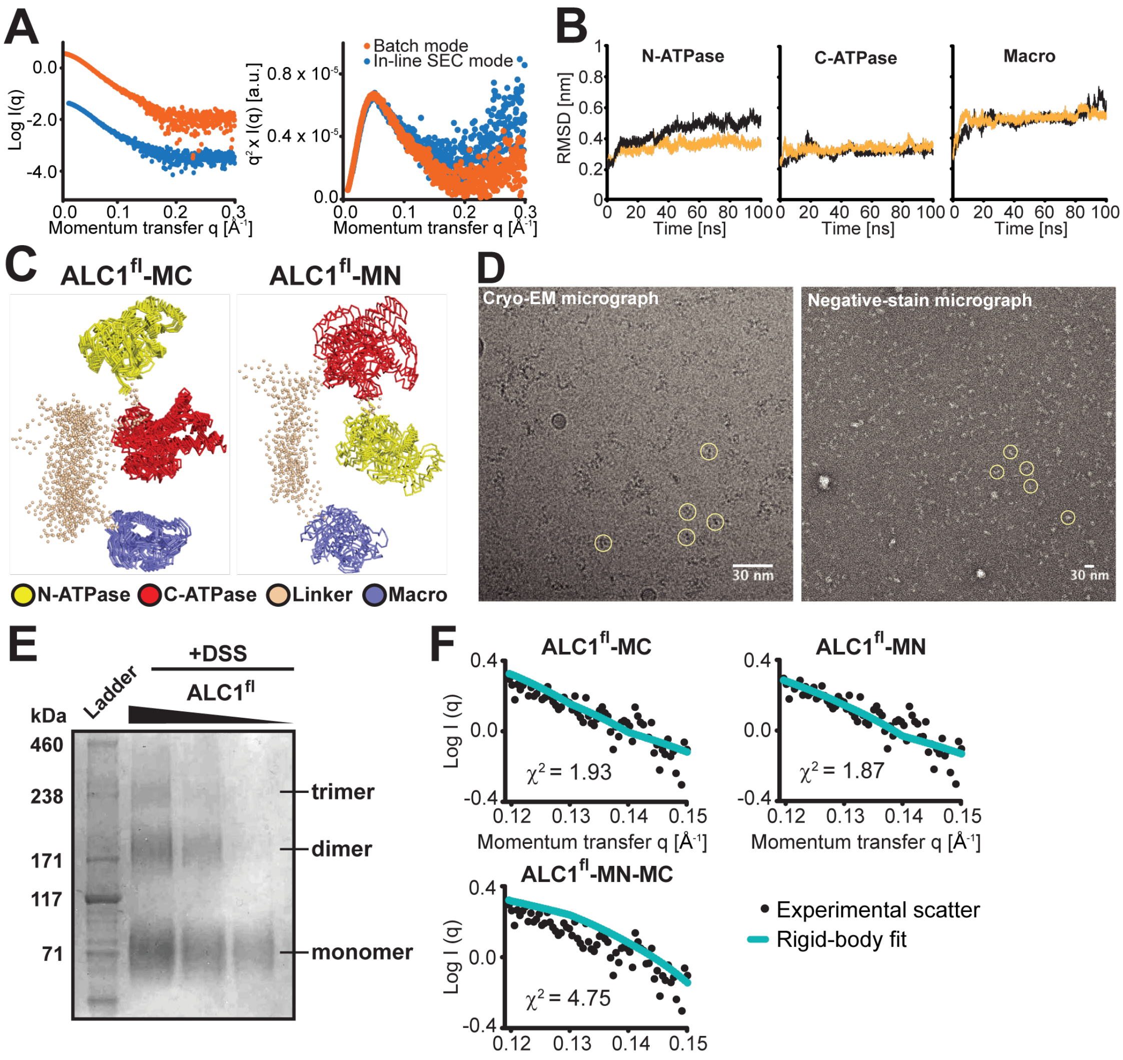


**Figure S2. Related to Figure 2. Additional analyses for structural modeling of ALC1$^{fl}$.**

(**A**) Left: Comparison of ALC1$^{fl}$ SAXS data measured in batch mode (orange) or with in-line size exclusion chromatography (blue). Curves are shifted along the y-axis by an arbitrary offset. Right: Kratky plots.

(**B**) Molecular dynamics simulations of the homology models show that they are stable and without unnatural energetic strain. The structural fluctuations during duplicate (black and orange) 100 ns simulations for the N-terminal ATPase lobe (left), the C-terminal ATPase lobe (middle), and the macro domain (right) shown as root mean squared deviation (RMSD) time traces. RMSD values represent deviations from the input homology model of the protein backbone, calculated after superimposing the individual structures with the backbone of the entire homology model.

(**C**) Overlay of individual rigid-body models from the dominant clusters of similar ALC1$^{fl}$-MC (left) or ALC1$^{fl}$-MN (right) structures.

(**D**) Micrographs from cryo- (left) and negative-stain (right) EM.

(**E**) SDS–PAGE analysis of ALC1$^{fl}$ cross-linking reactions with 1 mM DSS and 0.6, 0.3, and 0.15 mg/ml ALC1$^{fl}$ (from left to right). At dilute concentrations, only monomeric ALC1$^{fl}$ can be detected.

(**F**) Comparison of the experimental scattering recorded for ALC1$^{fl}$ with the theoretical scattering calculated for representative rigid-body models obtained with Macro:C-ATPase interdomain cross-links only (top left), Macro:N-ATPase interdomain cross-links only (top right), or both Macro:N-ATPase and Macro:C-ATPase interdomain cross-links (bottom) as restraints. When Macro:N-ATPase and Macro:C-ATPase cross-links are used as restraints at the same time, rigid-body modeling yielded a substantially worse fit to the experimental scattering data.

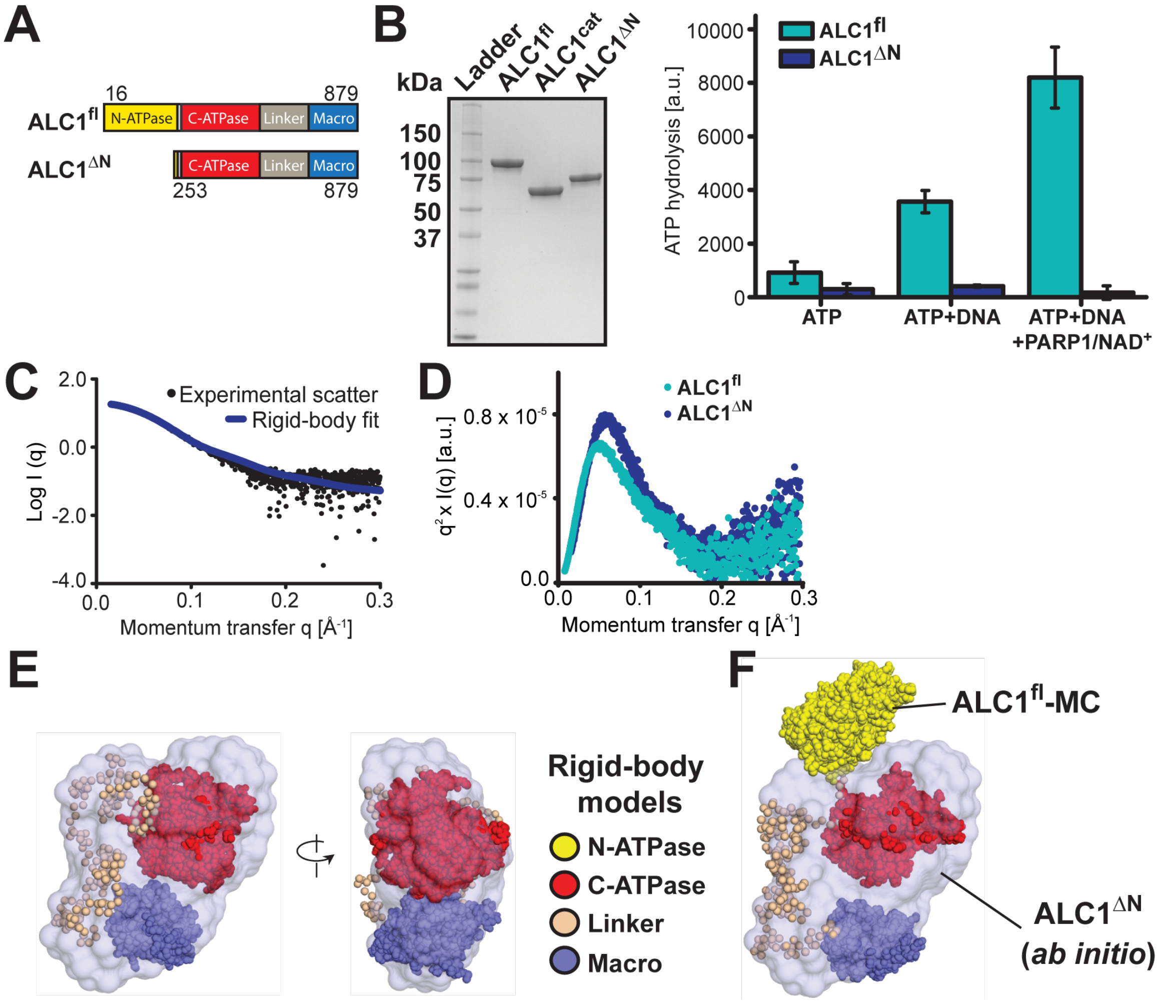


**Figure S3. Related to Figure 2. ATPase activity and SAXS-based modeling of ALC1$^{\Delta N}$.**
(**A**) Domain organization of ALC1$^{fl}$ (top) and ALC1$^{\Delta N}$ (bottom).
(**B**) Left: SDS–PAGE analysis of ALC1$^{\Delta N}$ (71.7 kDa), ALC1$^{fl}$ (98.8 kDa), and ALC1$^{cat}$ (68.6 kDa). The same gel as in Figure S1A (where the last lane was cropped from the image) is shown. Right: Comparison of ATPase activities of ALC1$^{\Delta N}$ (dark blue) and ALC1$^{fl}$ (teal). Where indicated, PARP1/NAD$^{+}$ and/or 239 bp dsDNA were added. Error bars represent SEM ($N = 3$ technical replicates).
(**C**) SAXS data for ALC1$^{\Delta N}$. The experimental data (black dots) are compared to the theoretical scattering curves (dark blue) calculated from the representative ALC1$^{\Delta N}$ rigid-body model shown (mean $\chi^2$ =2.0 for all 20 independent rigid body modeling trials).
(**D**) Comparison of the Kratky plots derived from batch-mode SAXS data collected with ALC1$^{fl}$ (teal) and ALC1$^{\Delta N}$ (dark blue).
(**E**) SAXS-derived structural model of ALC1$^{\Delta N}$. The structure obtained from rigid-body modeling with the C-terminal ATPase lobe, the linker, and the macro domain shown in red, light brown, and blue, respectively. The model is superimposed with a representative molecular envelope (gray) obtained from independent *ab initio* shape reconstruction.
(**F**) The ALC1$^{fl}$-MC structure is superimposed with the molecular envelope for ALC1$^{\Delta N}$ from (E).

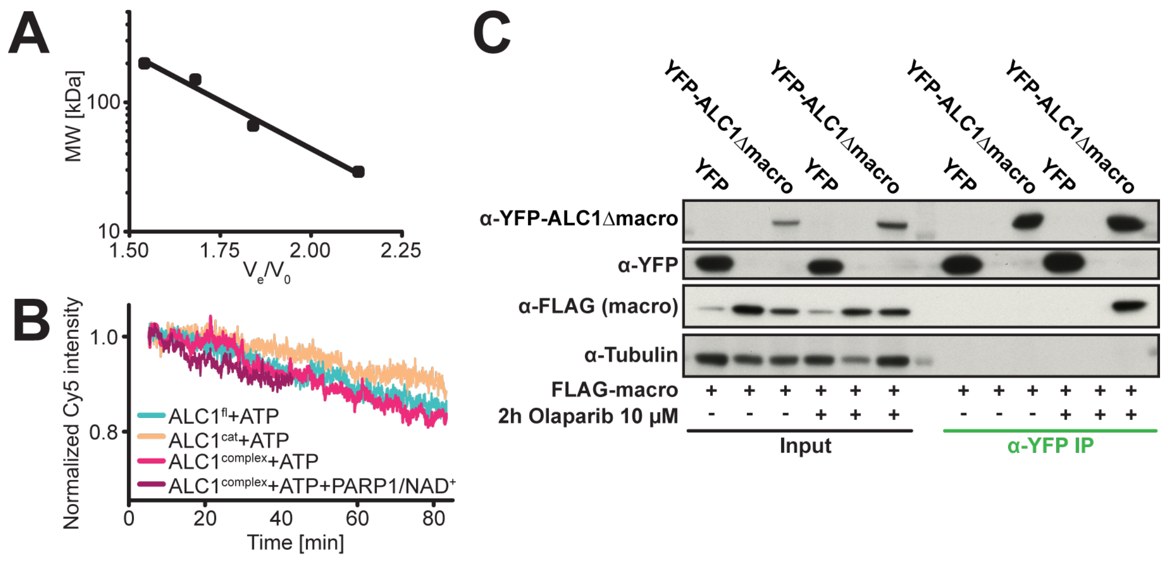


**Figure S4. Related to Figure 4. *In vitro* and *in vivo* complex formation.**

(**A**) Calibration curve for the analytical gel filtration shown in Figure 4A. β-amylase (200 kDa), alcohol dehydrogenase (150 kDa), bovine serum albumin (66 kDa), and carbonic anhydrase (29 kDa) were used as molecular weight standards to calibrate the Superdex 200 10/300 GL column.

(**B**) Nucleosome remodeling time course for $ALC1^{complex}$ in the presence of ATP (pink) compared to $ALC1^{fl}$ (teal) and $ALC1^{cat}$ (orange). Additionally, a remodeling time course for $ALC1^{complex}$ in the presence of ATP and $PARP1/NAD^{+}$ is shown. $PARP1/NAD^{+}$ releases the macro domain from the ATPase motor (Figure 4B), resulting in virtually the same remodeling kinetics as those observed with $ALC1^{cat}$. The curve for $ALC1^{fl}$ is the same as in Figure 1C.

(**C**) Co-immunoprecipitation (coIP) assay of U2OS cells transfected with plasmids expressing FLAG-tagged macro domain (FLAG-macro), YFP, or an YFP-tagged version of the ATPase motor (YFP-ALC1Δmacro). Cells were grown in the presence or absence of olaparib, as indicated. Cell lysates were immunoprecipitated with α-YFP (reciprocal to Figure 4C). The cell lysates (input) and immunoprecipitates were analyzed by immunoblotting with antibodies as indicated.

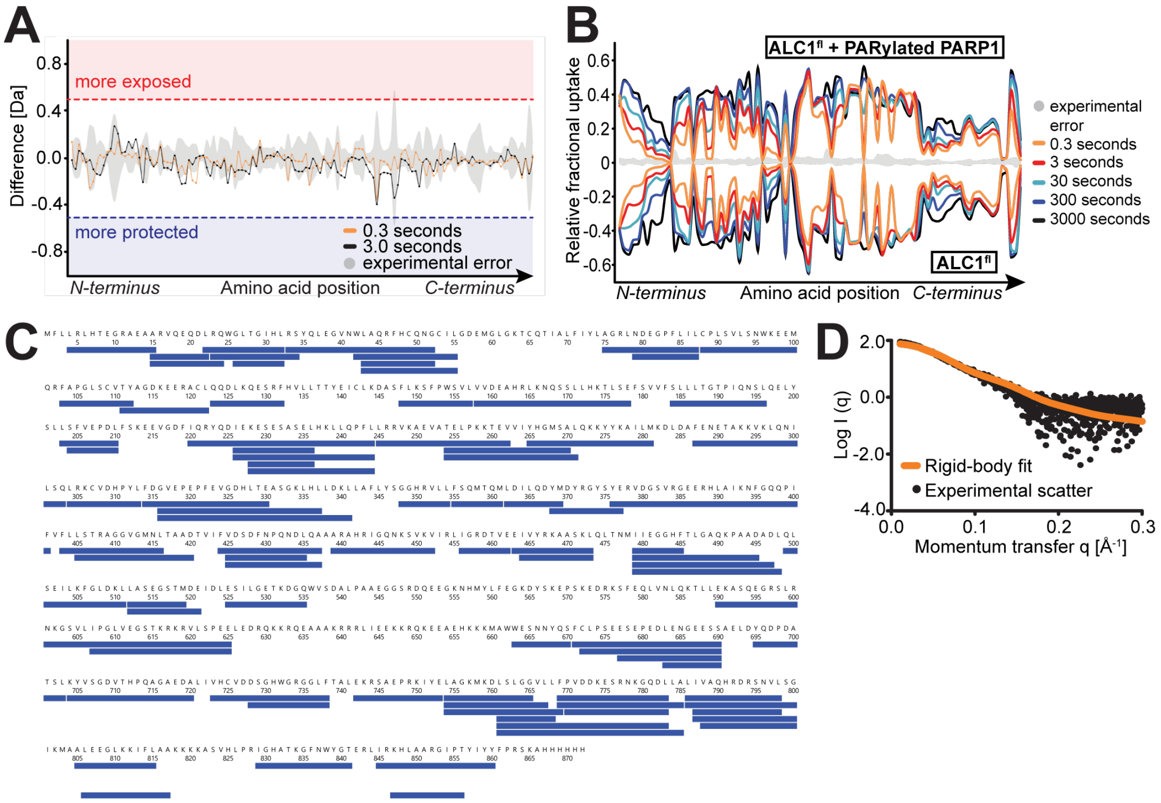


**Figure S5. Related to Figure 5. Additional HDX-MS analyses of ALC1$^{fl}$ and SAXS measurements with ALC1$^{cat}$.**

(**A**) Difference plot of hydrogen-deuterium exchange (HDX) data from ALC1$^{fl}$ in the presence and absence of non-PARylated PARP1. Changes beyond a threshold of ± 0.5 fall into areas of increased exposure (positive difference, shaded red) or protection (negative difference, shaded blue). The experimental error is shown in grey. Different line colors indicate the different time points at which deuterium labeling was carried out.

(**B**) Butterfly representation comparing the relative deuterium uptake of different regions of ALC1$^{fl}$ in the presence of PARylated PARP1 (top) with that of ALC1$^{fl}$ alone (bottom). The plot is shown for 5 different $D_2O$ incubation times (indicated by different colors) together with the experimental error from three replicates in grey. The difference plot shown in Figure 5A is derived from the data shown in the butterfly plot.

(**C**) Sequence coverage of ALC1$^{fl}$ for HDX experiments. The coverage is 75.2% with a total of 88 detected peptides.

(**D**) SAXS data measured with ALC1$^{cat}$ (black dots) and comparison with the theoretical scattering curves (orange) calculated from the rigid-body model shown in Figure 5C.

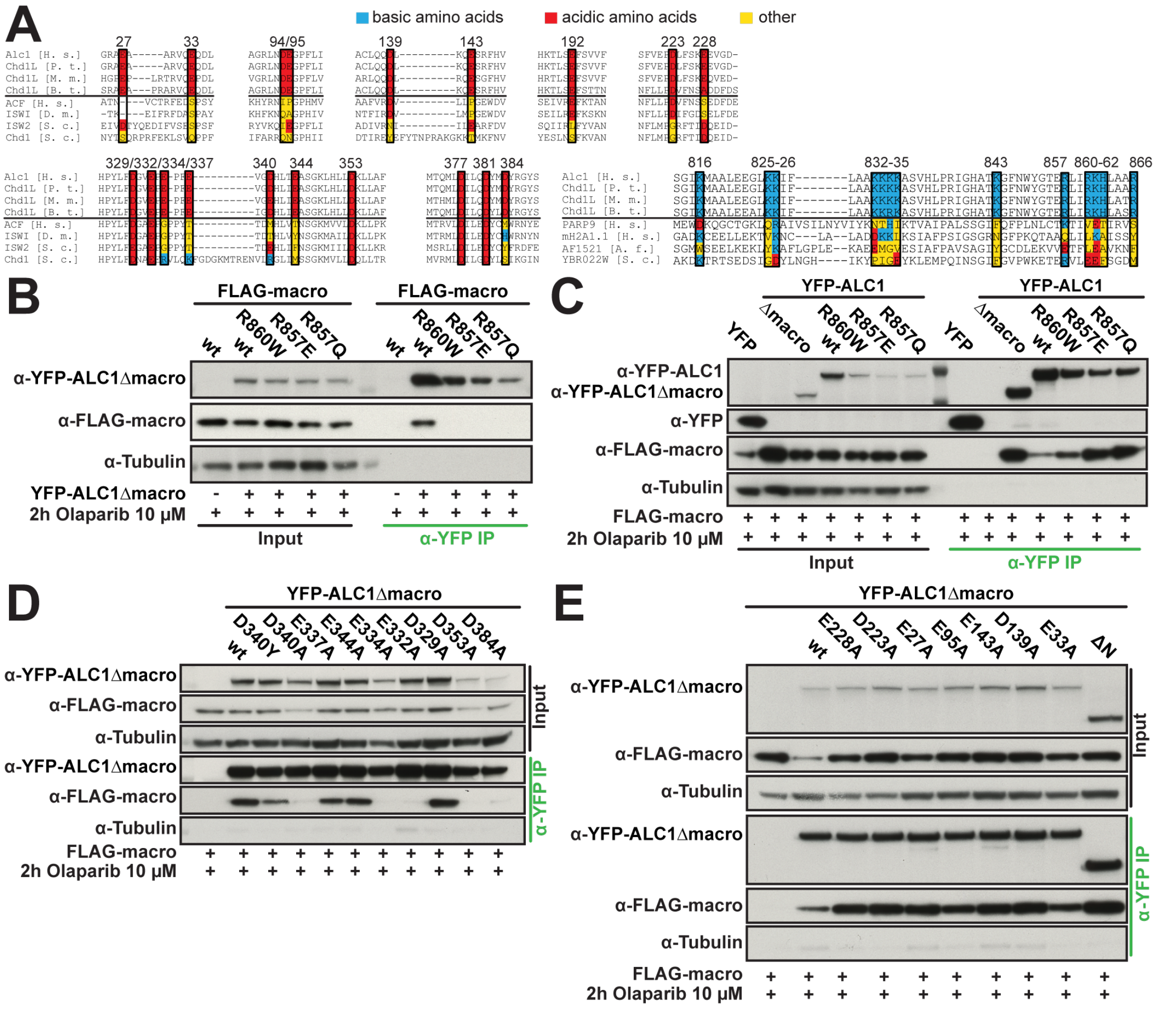


**Figure S6. Related to Figure 6. Sequence alignments and reciprocal co-IP assays.**

(**A**) Sequence alignments for the N- (top) or C-terminal (bottom left) ATPase lobes from ALC1/CHD1L and other remodelers and for the macro domains from ALC1/CHD1L and other proteins (bottom right). H.s. - *Homo sapiens*; P. t. - *Pan troglodytes*; M. m. - *Mus musculus*; B. t. - *Bos taurus*; D. s.: *Drosophila melanogaster*; S. c.: *Saccharomyces cerevisiae*; A. f. - *Archaeoglobus fulgidus*. Basic/acidic amino acids are highlighted in blue/red, respectively; others in yellow. The residue numbering corresponds to ALC1 [H. s.].

(**B**-**E**) CoIP assays with U2OS cells to probe macro domain-ATPase motor interactions. Cell lysates were immunoprecipitated with α-YFP (reciprocal to Figures 6B-E). As indicated, cells were grown and transfected with plasmids described for Figure 6. α-YFP immunoprecipitates were analyzed by immunoblotting with antibodies as indicated.

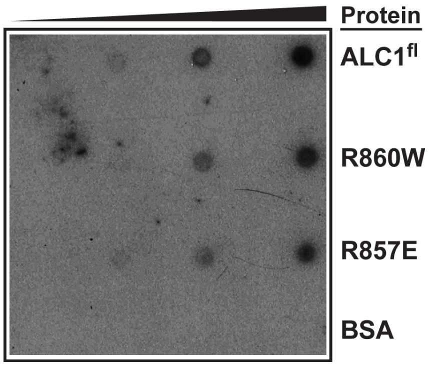


**Figure S7. Related to Figure 7. Binding of poly(ADP-ribose) (PAR) to wild-type ALC1$^{fl}$ and to ALC1$^{fl}$ mutants.**

2, 4, 8, and 16 pmol of ALC1$^{fl}$ wild-type, R860W, R857E, and BSA were dot-blotted onto a nitrocellulose membrane and incubated with $^{32}$P-labeled PAR. The same dot-blot as in Figure S1B (where the ALC1$^{fl}$ mutants R860W and R857E were cropped from the image) is shown.

| Peptides | Residue 1 | Residue 2 | Id-Score |
|---|---|---|---|
| KTEVVIYHGMSALQK-RVKAEVATELPK-a1-b3 | 273 | 263 | 32.67 |
| KTEVVIYHGMSALQKK-VKAEVATELPK-a1-b2 | 273 | 263 | 29.01 |
| KTEVVIYHGMSALQK-VKAEVATELPK-a1-b2 | 273 | 263 | 28.71 |
| ACLQQDLKQESR-IGQNKSVK-a8-b5 | 141 | 462 | 29.58 |
| LKNQSSLLHK-IGQNKSVK-a2-b5 | 180 | 462 | 27.81 |
| YQDIEKESESASELHK-KAASK-a6-b1 | 242 | 482 | 30.07 |
| YQDIEKESESASELHK-SVKVIR-a6-b3 | 242 | 465 | 33.00 |
| YQDIEKESESASELHK-IGQNKSVK-a6-b5 | 242 | 462 | 30.08 |
| VKAEVATELPK-SVKVIR-a2-b3 | 263 | 465 | 30.81 |
| RVKAEVATELPK-SVKVIR-a3-b3 | 263 | 465 | 29.08 |
| GGLFTALEKR-LKNQSSLLHK-a9-b2 | 757 | 180 | 28.60 |
| NKGQDLLALIVAQHR-ACLQQDLKQESR-a2-b8 | 793 | 141 | 24.49 |
| NKGQDLLALIVAQHR-LKNQSSLLHK-a2-b2 | 793 | 180 | 28.82 |
| IGHATKGFNWYGTER-ACLQQDLKQESR-a6-b8 | 848 | 141 | 29.58 |
| FAPGLSCVTYAGDKEER-KHLAAR-a14-b1 | 130 | 861 | 26.65 |
| FAPGLSCVTYAGDKEER-NKGQDLLALIVAQHR-a14-b2 | 130 | 793 | 16.52 |
| FAPGLSCVTYAGDKEER-GGLFTALEKR-a14-b9 | 130 | 757 | 26.14 |
| ACLQQDLKQESR-GGLFTALEKR-a8-b9 | 141 | 757 | 29.92 |
| LKNQSSLLHK-KHLAAR-a2-b1 | 180 | 861 | 27.95 |
| YQDIEKESESASELHK-KHLAAR-a6-b1 | 242 | 861 | 28.33 |
| YQDIEKESESASELHK-IGHATKGFNWYGTER-a6-b6 | 242 | 848 | 29.70 |
| YQDIEKESESASELHK-NKGQDLLALIVAQHR-a6-b2 | 242 | 793 | 18.20 |
| YQDIEKESESASELHK-GGLFTALEKR-a6-b9 | 242 | 757 | 24.01 |
| VKAEVATELPK-KHLAAR-a2-b1 | 263 | 861 | 26.30 |
| VKAEVATELPK-GGLFTALEKR-a2-b9 | 263 | 757 | 25.92 |
| GGLFTALEKR-IGQNKSVK-a9-b5 | 757 | 462 | 28.39 |
| GGLFTALEKR-SVKVIR-a9-b3 | 757 | 465 | 31.50 |
| SNVLSGIKMAALEEGLKK-IGQNKSVKVIR-a8-b8 | 816 | 465 | 23.75 |
| IGHATKGFNWYGTER-SVKVIR-a6-b3 | 848 | 465 | 30.79 |
| IGQNKSVK-KIYELAGK-a5-b1 | 462 | 764 | 24.41 |

**Table S1. Related to Figure 2. List of cross-linked peptides.**
Inter-domain cross-links between the N-terminal and the C-terminal ATPase lobes, between the macro domain and the N-terminal ATPase lobe, and between the macro domain and the C-terminal ATPase lobe are shown in orange, green, and purple, respectively. Only cross-links with an Id score (Walzthoeni et al., 2012) of > 15 were included.

| Construct/Mutation | Forward Primer | Reverse Primer |
|---|---|---|
| ALC1Δmacro | TAAACAAAGTGGTGATATCAAAC | TTCCTTTTGCCTCTTCTTC |
| ALC1ΔN | GAGGTAGCTACAGAGCTTC | GAAGCCTGCTTTTTTGTAC |
| D94A | CCAGCTGGAGgccGAGTAAACTG | TAAGAGCGTAGGTGAATC |
| E228A | CTTTTCCAAGgccGAGGTGGGAG | AGATCAGGCTCCACAAAAC |
| D223A | TGTGGAGCCTgccCTCTTTTCCAAG | AAACTGAGGAGGGAGTAG |
| E27A | GGGCCGAGCCgccGCGGCGCGGG | GCCGCCTCGGCTCGGCCC |
| E192A | GACCTTGTCAgccTTCTCAGTAGTCTTCAGTC | TTATGCAGCAGGGAGCTT |
| E95A | ATTAAATGATgccGGGCCATTTCTG | CTTCCTGCCAAATAAATG |
| E143A | CCTGAAACAGgccTCACGTTTTCATGTGCTAC | TCTTGCTGAAGGCAGGCT |
| D139A | CCTTCAGCAAgccCTGAAACAGG | CAGGCTCTTTCCTCCTTG |
| E33A | GCGGGTGCAGgccCAGGACTTACGGCAGTGGG | GCCGCCTCGGCTCGGCCC |
| D340Y | TGAAGTTGGAtacCACCTGACTGAGG | AAAGGCTCCGGCTCCACA |
| D340A | TGAAGTTGGAgccCACCTGACTG | AAAGGCTCCGGCTCCACA |
| E337A | GGAGCCTTTTgccGTTGGAGACC | GGCTCCACACCATCAAAC |
| E344A | CCACCTGACTgccGCTAGTGGGAAG | TCTCCAACTTCAAAAGGC |
| E334A | TGTGGAGCCGgccCCTTTTGAAG | CCATCAAACAAATATGGGTG |
| E332A | TGATGGTGTGgccCCGGAGCCTT | AACAAATATGGGTGATCCAC |
| D329A | ATATTTGTTTgccGGTGTGGAGCCG | GGGTGATCCACACACTTTC |
| D353A | TCACCTGCTGgccAAGCTACTAGC | AGCTTCCCACTAGCCTCA |
| D384A | GACTATATGGgccACAGAGGCTACAG | TTGGAGAATATCCAACATC |
| R857Q | TGGTACTGAGcaaCTTATTCGGAAAC | TACCAGTTAAAACCTTTCGTG |
| R857E | TGGTACTGAGgaaCTTATTCGGAAAC | TACCAGTTAAAACCTTTCG |
| R860W | GCGACTTATTtggAAACATCTGG | TCAGTACCATACCAGTTAAAAC |

**Table S2. Related to STAR Methods. DNA oligonucleotides used to create ALC1 constructs for *in vivo* analysis.**
